# Relative Contributions of Various Cellular Mechanisms to Loss of Algae during Cnidarian Bleaching

**DOI:** 10.1371/journal.pone.0152693

**Published:** 2016-04-27

**Authors:** Tamaki Bieri, Masayuki Onishi, Tingting Xiang, Arthur R. Grossman, John R Pringle

**Affiliations:** 1 Department of Genetics, Stanford University School of Medicine, Stanford, California, United States of America; 2 Department of Biology, Stanford University, Stanford, California, United States of America; 3 Department of Plant Biology, Carnegie Institution for Science, Stanford, California, United States of America; Northeastern University, UNITED STATES

## Abstract

When exposed to stress such as high seawater temperature, corals and other cnidarians can bleach due to loss of symbiotic algae from the host tissue and/or loss of pigments from the algae. Although the environmental conditions that trigger bleaching are reasonably well known, its cellular and molecular mechanisms are not well understood. Previous studies have reported the occurrence of at least four different cellular mechanisms for the loss of symbiotic algae from the host tissue: *in situ* degradation of algae, exocytic release of algae from the host, detachment of host cells containing algae, and death of host cells containing algae. The relative contributions of these several mechanisms to bleaching remain unclear, and it is also not known whether these relative contributions change in animals subjected to different types and/or durations of stresses. In this study, we used a clonal population of the small sea anemone *Aiptasia*, exposed individuals to various precisely controlled stress conditions, and quantitatively assessed the several possible bleaching mechanisms in parallel. Under all stress conditions tested, except for acute cold shock at 4°C, expulsion of intact algae from the host cells appeared to be by far the predominant mechanism of bleaching. During acute cold shock, *in situ* degradation of algae and host-cell detachment also became quantitatively significant, and the algae released under these conditions appeared to be severely damaged.

## Introduction

**Fig 1:**
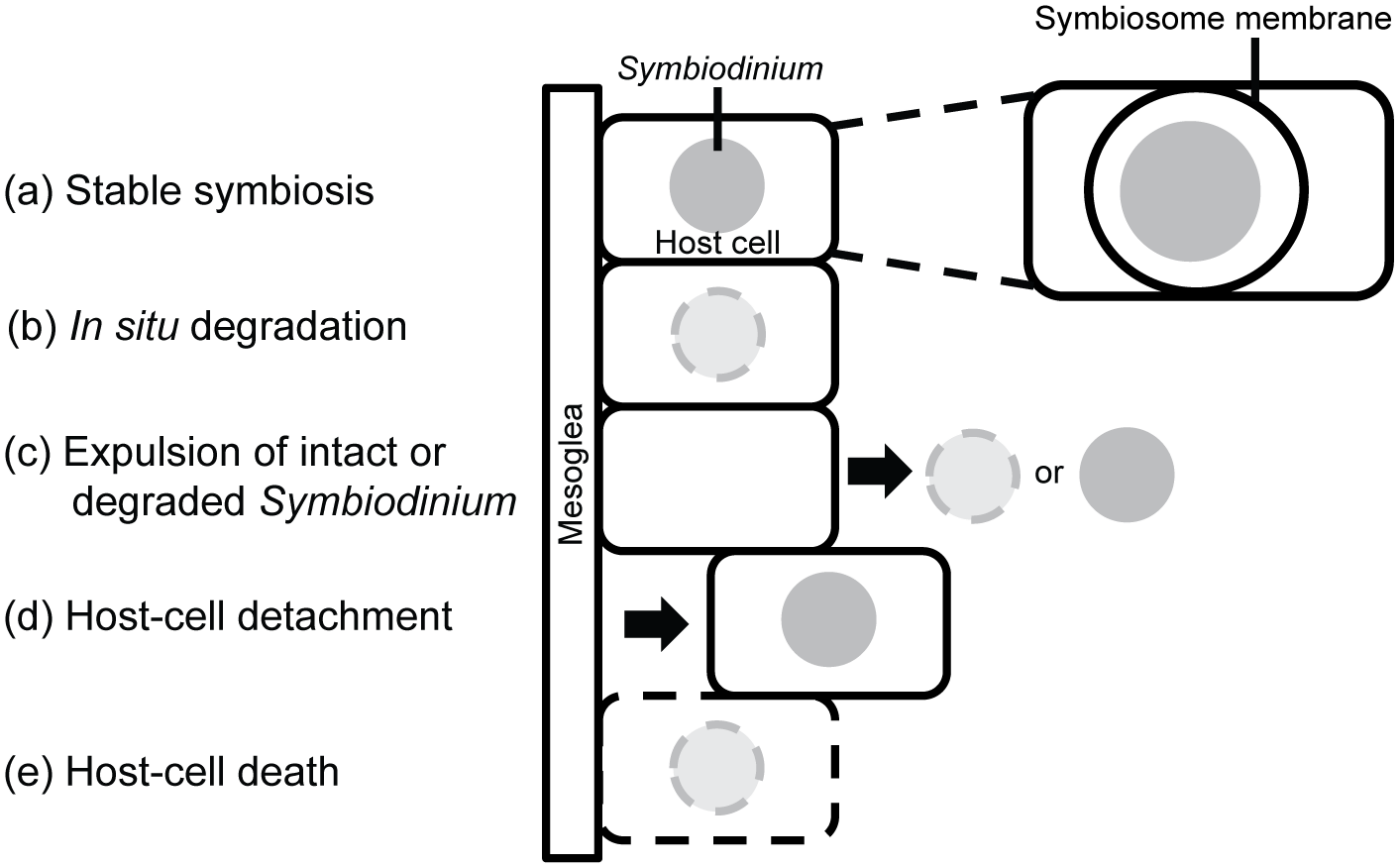
Four possible cellular mechanisms of cnidarian bleaching under stress. During stable symbiosis (a), algae reside within gastrodermal cells of the cnidarian host and are surrounded by the host-derived symbiosome membrane. Under stress, algae could be lost (b) by *in situ* degradation (involving fusion of the symbiosome with lysosomes, autophagy, and/or a cell-death reaction of the algae themselves), (c) by expulsion of healthy and/or degraded algae, (d) by detachment of algae-containing host cells, (e) by death of algae-containing host cells through apoptosis or necrosis, or by some combination of these mechanisms.

Coral reefs are of enormous ecological, economic, and aesthetic importance [1], but their health around the world has declined rapidly in recent years due to anthropogenic stresses such as pollution, overfishing, rising ocean temperatures, and falling ocean pH [2]. Most of the energy used by corals for growth and reef deposition comes from photosynthetic dinoflagellates of the genus *Symbiodinium*, which live in specialized endosomes ("symbiosomes") within the gastrodermal cells of the host (Fig 1a) and provide it with fixed carbon in exchange for shelter and inorganic nutrients [3,4]. Under stress, corals and other symbiotic cnidarians can lose coloration ("bleach") because of the loss of photosynthetic pigments from the algae, the loss of algae from the host tissue, or both; if prolonged, bleaching can lead to death of the host [5]. Despite the biological interest and practical importance of bleaching, its cellular and molecular mechanisms remain very poorly understood [4–6].

In this study, we have focused on the cellular mechanisms of algal loss during bleaching. At least four general mechanisms for algal loss can be imagined, and indeed all four have been reported to occur. First, algal cells might be degraded *in situ* (Fig 1b). The mechanism might involve acidification of the symbiosome lumen and fusion with lysosomes containing low-pH-active hydrolases [7], autophagic engulfment and digestion of the entire symbiosome [8,9], or a cell-death reaction of the algae themselves. *In situ* degradation of algae has been reported in corals under thermal stress during natural bleaching events [10,11] and upon exposure of corals or anemones to temperature and/or light stress in the laboratory [12–17]. Second, intact or degraded algae might be expelled from the host cells by exocytosis or a related mechanism (Fig 1c). Such expulsion has been reported in corals during natural thermal bleaching events [11] and when corals or anemones were exposed to temperature and/or light stress in the laboratory [18–23]. Third, host cells containing algae might be detached from the gastrodermal layer and released into the gut and thence the environment (Fig 1d). Such detachment has been reported both during a natural thermal bleaching event [11] and when corals or anemones were exposed to heat stress, cold stress, or caffeine in the laboratory [24,25]. Fourth, host cells containing algae might die in place by either a programmed, apoptotic mechanism or by necrosis (Fig 1e), and numerous studies have reported such host-cell death during thermally induced bleaching in both corals and anemones [13,14,26–32]. Reactive oxygen species released by the algae, host- and/or symbiont-produced nitric oxide, activation of host innate-immune responses, and degradation of host mitochondria upon exposure to stress have all been suggested to be involved in inducing host-cell apoptosis [6,28,33,34].

This bewildering variety of conflicting reports may reflect, at least in part, the fact that different investigators have studied different species, or different genotypes of the same species, using a variety of different stress conditions and assays, so that it is difficult to compare directly the results of different studies. In addition, to our knowledge, none of the previous studies has attempted to evaluate quantitatively the relative contributions of the various possible mechanisms to bleaching in a single type of organism under a variety of stress conditions. Moreover, many of the previous studies have worked with calcifying corals, which pose many challenges for performing such quantitative cell-biological studies.

We have attempted to address these issues using the small sea anemone *Aiptasia*, which is symbiotic with *Symbiodinium* strains similar to those found in corals and is emerging as an increasingly powerful model organism for the study of cnidarian-dinoflagellate symbiosis [35–41]. Among the experimental advantages of *Aiptasia* are that large clonal populations can be obtained and that it can be maintained indefinitely in an aposymbiotic (dinoflagellate-free) state [42–44]. We subjected a clonal population of anemones to a variety of precisely controlled stress conditions and assessed the relative contributions of the various possible bleaching mechanisms. We found that under all conditions (with the possible exception of acute cold shock), expulsion of algae by the host cells appeared to be the predominant mechanism of bleaching. Although some apoptosis appeared to occur as part of the immediate response to thermal stress, this process did not appear to play a significant role in bleaching, and host-cell detachment and *in situ* degradation of algae appeared to play significant roles only during acute cold shock at 4°C.

## Materials and Methods

### Organisms and standard culture conditions

Anemones of the clonal *Aiptasia* line CC7 [45] were used in all experiments; the animals were either symbiotic with their endogenous clade A *Symbiodinium* or had been rendered aposymbiotic as described previously [36,46]. Artificial seawater (ASW) was made by mixing Coral Pro Salt (Red Sea, Houston, TX) with deionized water to a concentration of ~34 ppt, pH 8 ± 0.1. Animals were routinely maintained ("standard conditions") in ASW at 27°C and 25 μmol photons m^-2^ s^-1^ white fluorescent light (Philips ALTO II 25W bulbs) on a 12L:12D regimen in polycarbonate tubs in a growth chamber; each tub contained 13–50 animals in ~1 L of ASW. Animals were fed twice weekly with freshly hatched *Artemia* nauplii, and the ASW was replaced four times per week.

The clonal, axenic *Symbiodinium* strain SSA01 was isolated from CC7 anemones essentially as described previously for the isolation of strain SSB01 from H2 anemones [46]. Several tests confirmed that the cultures were indeed axenic (S4 Fig), and sequencing of the *cp23S* and *ITS2* regions [47] confirmed that SSA01 is a member of Clade A, subclade A4, and thus can be assigned to species *S*. *linucheae* [48]. SSA01 cells were routinely grown at 27°C in Daigo’s IMK medium (Wako Pure Chemicals, Osaka, Japan) supplemented with both casein hydrolysate (4 g/L) and glucose (5 g/L) [46] at 10 μmol photons m^-2^ s^-1^ from 35-W G.E. Cool White fluorescent bulbs on a 12L:12D regimen.

### Stress conditions used to induce bleaching

A variety of temperature and light conditions was used to apply stress leading to bleaching (Table 1). ASW was pre-heated or pre-chilled to the temperature of each stress condition and added to tubs containing anemones. For all stress conditions except cold shock, the tubs containing ~1 L of ASW and 20–80 anemones were then placed in a growth chamber set to the indicated temperature and light level on a 12L:12D regimen and left under these conditions for the duration of the experiment (as indicated in the figure and table legends). For cold shock, the tubs containing ~1 L of ASW and 16–60 anemones were placed in a refrigerator at 4°C in the dark near the beginning of the normal light phase. After 4 h, the tubs were returned to standard culture conditions (27°C, 25 μmol photons m^-1^ s^-2^) without an ASW change (thus, a gradual warm-up) and maintained that way for 24 h before collecting animals or expelled algae for analysis. During the stress experiments, the animals were not fed (the last feeding was 4 d before the beginning of each experiment, with a last ASW change immediately before the experiment began), and the ASW was replaced daily both for the animals exposed to stress and for the control animals under standard culture conditions. Although there was considerable animal-to-animal variation within any one tub (see Results), we also considered the possibility that there might be systematic differences between tubs (animal-to-animal communication; different bacterial biofilms; etc.) and so considered as "independent experiments" only those involving tubs of animals that had been maintained separately for at least one month. Importantly, under all stress conditions and experimental durations used in this study, 100% of the animals survived and indeed seemed to be grossly normal in behavior (except for the losses of algae).

**Table 1:** Temperature and light treatments used to apply stress.

Stress condition	Temperature [°C]	Light [μmol photons m^-2^ s^-1^]
Heat	34	25
Heat and light	34	150
High light	27	500
Cold	18	25
Cold shock	4	0 ^a^

^a^ During the 4 h cold-shock treatment; animals were then returned to standard culture conditions (27°C, 25 μmol photons m^-2^ s^-1^).

### Caspase-inhibitor treatments

The caspase inhibitor Ac-DEVD-CHO (Promega, Cat. No. G5961; provided as a 10 mM stock solution in DMSO) was used; it should inhibit *Aiptasia* caspases similar to mammalian caspases 3, 7, and 8 [49,50] (S1 Fig). Anemones were incubated with 2 μM inhibitor in ~100 ml of ASW (a 5000-fold dilution of the stock solution) for 1 h at 27°C in the dark, then moved without a water change to a growth chamber at 34°C and 150 μmol photons m^-2^ s^-1^ on a 12L:12D regimen, where the ASW gradually warmed to the new temperature. The ASW containing caspase inhibitor was replaced daily after pre-warming the ASW to 34°C; the controls (ASW with no additives and ASW with 0.02% DMSO) had water changes on the same schedule.

### Measurements of caspase activity

Caspase activity was determined using the ApoAlert Fluorescent Caspase-3 Kit (Clontech Laboratories, Cat. No. 630215), which should detect the activities of *Aiptasia* caspases similar to mammalian caspases 3, 7, and (probably) 8 (S1 Fig) [49,50]. Individual fresh (not previously frozen) anemones were homogenized in 300 μl lysis buffer (from the kit) using a PowerGen 125 rotor stator homogenizer (Fisher Scientific) [51]. Algal cells [which are not disrupted by the homogenizer [35]] were removed by centrifugation at 13,000 rpm for 10 min at 4°C in a table-top centrifuge, and the supernatants were frozen for later analysis. For analysis, the extracts were thawed, mixed 1:1 with 2X Reaction Buffer containing dithiothreitol and DMSO, and incubated with the substrate Ac-DEVD-AFC at 37°C for 1 h according to the manufacturer’s instructions. Fluorescence (excitation at 400 nm, emission at 505 nm) readings were then taken using an Infinite Pro spectrophotometer (Tecan, Männedorf, Switzerland). The relative fluorescence units obtained for each sample were normalized to the total protein present in that sample, as determined using the Pierce BCA Protein Assay (Thermo Scientific) according to the manufacturer’s instructions; absorbances were read at 562 nm using the Infinite Pro spectrophotometer.

### Determination of algal cell numbers

The numbers of algae in animals and (where appropriate) in the ASW from the tubs were determined as described previously [51]. Briefly, animals were frozen and subsequently thawed and homogenized using a PowerGen 125 rotor stator homogenizer (Fisher Scientific). Algal cell numbers were then determined using a Guava easyCyte HT 2 laser flow cytometer with the InCyte v2.7 software (EMD Millipore), which allows highly precise counts (plus/minus ~2% [51]). ASW containing algae released from anemones was passed through a 27-gauge syringe 10 times in order to disrupt boluses of algae and allow effective counting of single cells with the flow cytometer. Protein concentrations of the homogenates were determined using the BCA assay (see above); this method has precision comparable to that of the Guava [51].

### Confocal microscopy

Anemones were fixed in 4% formaldehyde in ASW at 4°C overnight and then stored at room temperature until further processing upon arrival at the Histology Laboratory, Institute of Neuroscience, University of Oregon. For processing, the anemones were embedded in a mixture of 1% low-melting-point agarose, 0.5% agar, and 5% sucrose, and sections of ~16 μm thickness were cut using a Leica CM3050 S cryostat. Samples of material released by the anemones into the ASW (*i*.*e*., algae plus any gastrodermal cells containing algae) were fixed in 4% formaldehyde in ASW at 4°C overnight. Sections and samples of the released material were rinsed three times with phosphate buffered saline (PBS), stained with 300 nM DAPI (4',6-diamidino-2-phenylindole) (Invitrogen, Cat. No. D1306) in ASW for 1 h at room temperature, rinsed three times with PBS, and mounted on glass slides with VECTASHIELD Hard Set Mounting Medium (Vector Laboratories, Cat. No. H-1400). The cells were then imaged using a 63X/NA 1.4 oil-immersion lens on a SP5 AOBS or SP8X (white-light laser) point-scanning confocal microscope (Leica Microsystems). DAPI was excited at 405 nm with emission collected at 428–470 nm, and the red auto-fluorescence of the algae was excited at 488 nm with emission collected at 628–726 nm. The Leica Application Suite Advanced Fluorescence software was used to control the microscope, and ~20 optical sections per field of view were collected with a step size of 0.4 μm. Images were analyzed with the open-source software Fiji. Briefly, DAPI-stained nuclei were identified by applying the threshold “moments” and using the “analyze particles” function with the following settings: particle size 1–10 μm^2^, circularity, 0.5–1. The outlines of the nuclei were overlaid with the corresponding images showing algal autofluorescence. Each individual nucleus was followed through the *z*-stack and scored manually as either being inside or outside of an algal cell.

### Electron microscopy

Anemones and released algae were fixed in 4% paraformaldehyde + 4% glutaraldehyde in ASW first at RT for 1 h, then at 4°C overnight, and then rinsed three times with 0.1 M sodium cacodylate buffer, pH 6.8. Released algae were then embedded in 10% gelatin in the cacodylate buffer. Anemones and embedded algae were then post-fixed in 1% OsO4 in distilled water for 2 h at 4°C, washed three times with distilled water, incubated in 1% uranyl acetate in distilled water at 4°C overnight, and dehydrated through a graded series of increasing ethanol concentrations. After replacement of ethanol with acetonitrile, samples were embedded in EMbed-812 resin (Electron Microscopy Sciences, Cat. No. 14120). Sections of ~70 nm were cut using a Leica Ultracut S microtome, collected on slots or 100-mesh copper grids (Electron Microscopy Sciences) that had been coated with formvar and/or evaporated carbon to stabilize the sections, and stained for 30 sec in 1:1 3% uranyl acetate and 50% acetone, followed by 0.2% lead citrate for 3 min. Sections were then imaged at 120 kV using a JEM-1400 transmission electron microscope (JEOL) equipped with a Gatan Orius 4k X 4k digital camera. The algae in each section were scored manually as being "intact" [algal cell adjacent or close to the symbiosome membrane, and at least two subcellular structures (nucleus, chloroplast, and pyrenoid) visible and normal in appearance], "degrading" (algal cell significantly separated from the symbiosome membrane and all subcellular structures either not visible or shriveled up and without normal appearance), or ambiguous (not clearly assignable to either of the other categories; typically either algal cell not separated from the symbiosome membrane but subcellular structures not clearly visible, or algal cell separated from symbiosome membrane but subcellular structures still visible and recognizable). Examples illustrating each category are provided in the figure showing these data. In examining the host gastrodermal cells, we performed a qualitative evaluation of the numbers, spatial distribution, and structural appearance of intracellular membranous organelles and of the relationship of the cells to the adjacent mesoglea.

### Measurement of *F*_*v*_*/F*_*m*_

*F*_*v*_*/F*_*m*_ measurements for SSA01 cultures and released algae were performed on cell suspensions (~2x10^6^ cells ml^-1^) using a Dual PAM-100 fluorometer (Heinz Walz) as described previously [52]. *F*_*v*_*/F*_*m*_ measurements on CC7 anemones were performed using a JTS10 spectrophotometer (BioLogic). Both cell suspensions and anemones were incubated in the dark for 40 min before measurements were made.

### Statistical analyses

**Fig 2:**
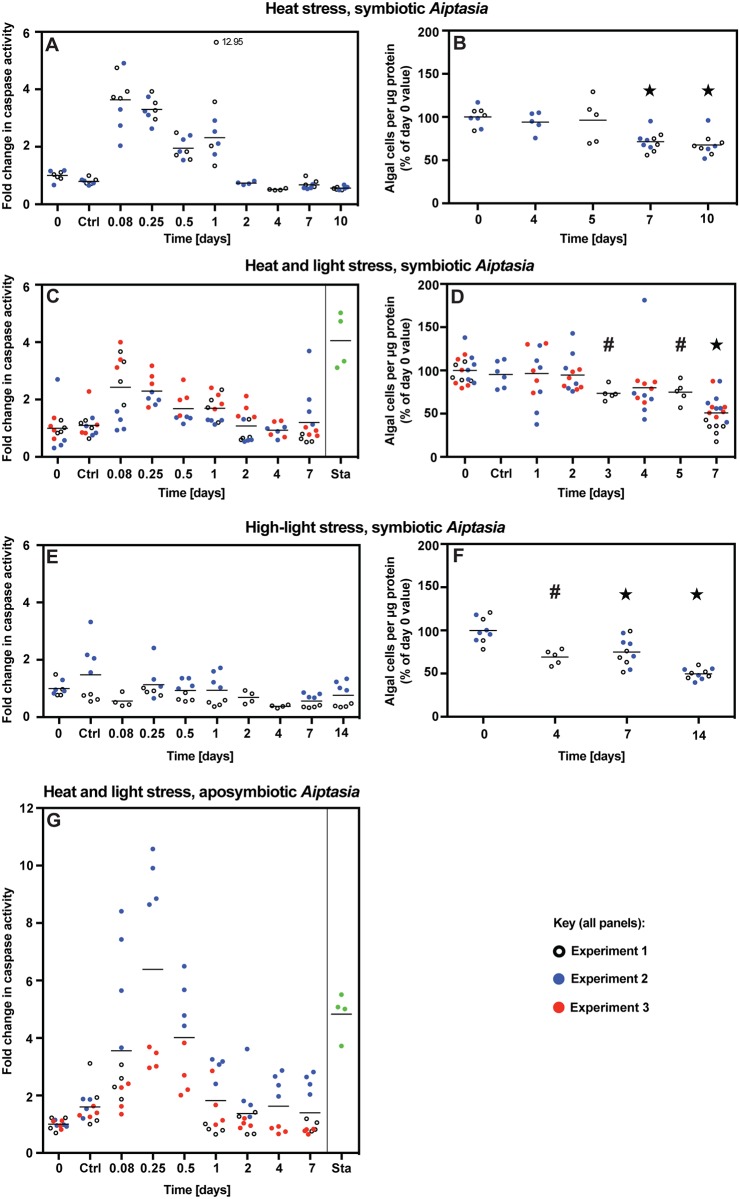
Lack of correlation between the timing of apoptosis (as judged by caspase activation) and that of bleaching in *Aiptasia* under heat and/or light stress. (A-F) Symbiotic anemones previously acclimated under standard culture conditions (27°C, 25 μmol photons m^-2^ s^-1^) were exposed to (A,B) heat stress alone (34°C, 25 μmol photons m^-2^ s^-1^), (C,D) heat and light stress (34°C, 150 μmol photons m^-2^ s^-1^), or (E,F) light stress alone (27°C, 500 μmol photons m^-2^ s^-1^) for the durations indicated, and samples were taken from each tub for the determination of caspase activities (A,C,E) and numbers of algae per unit protein (B,D,F) in homogenates of individual animals (see Materials and Methods). Two (A,B,E,F) or three (C,D) independent experiments (i.e., animals in separate tubs—see Materials and Methods) were performed, and caspase activities and algal counts were determined for 4–5 individual animals per time point in each experiment. The values for all individual animals are shown using different symbols for the independent experiments (as indicated). [Note that the experiments of Figs 3A and 5A (samples with no caspase inhibitor) provide additional independent replications for the most important time points in regard to heat and light stress.] For each panel, values were normalized to the mean values for all animals from all experiments before exposure to the stress conditions at 0 d (set at 1.0 for caspase or 100% for algal counts). The mean value for all animals at each time-point is indicated by a horizontal line; note that one animal in *A*, Experiment 1, appeared to give an artifactually high caspase activity (12.96 normalized value) and was excluded in calculating the mean activity for that time-point. Ctrl, anemones left at 27°C, 25 μmol photons m^-2^ s^-1^, for the duration of the experiment; Sta, anemones incubated overnight (27°C; 12 h of dark bracketed by ~1-h periods at 25 μmol photons m^-2^ s^-1^) with 3 μM staurosporine to induce apoptosis (this test was performed in just one of the three replicate experiments for C). (G) Aposymbiotic animals were analyzed as for symbiotic animals in C; three independent experiments were performed. In panels B, D, and F, mean values that appear significantly different (*P* ≤ 0.005) from the day-0 means are indicated by a ★ (grouped ANOVA) or # (unpaired *t* test) (see Materials and Methods).

**Fig 3:**
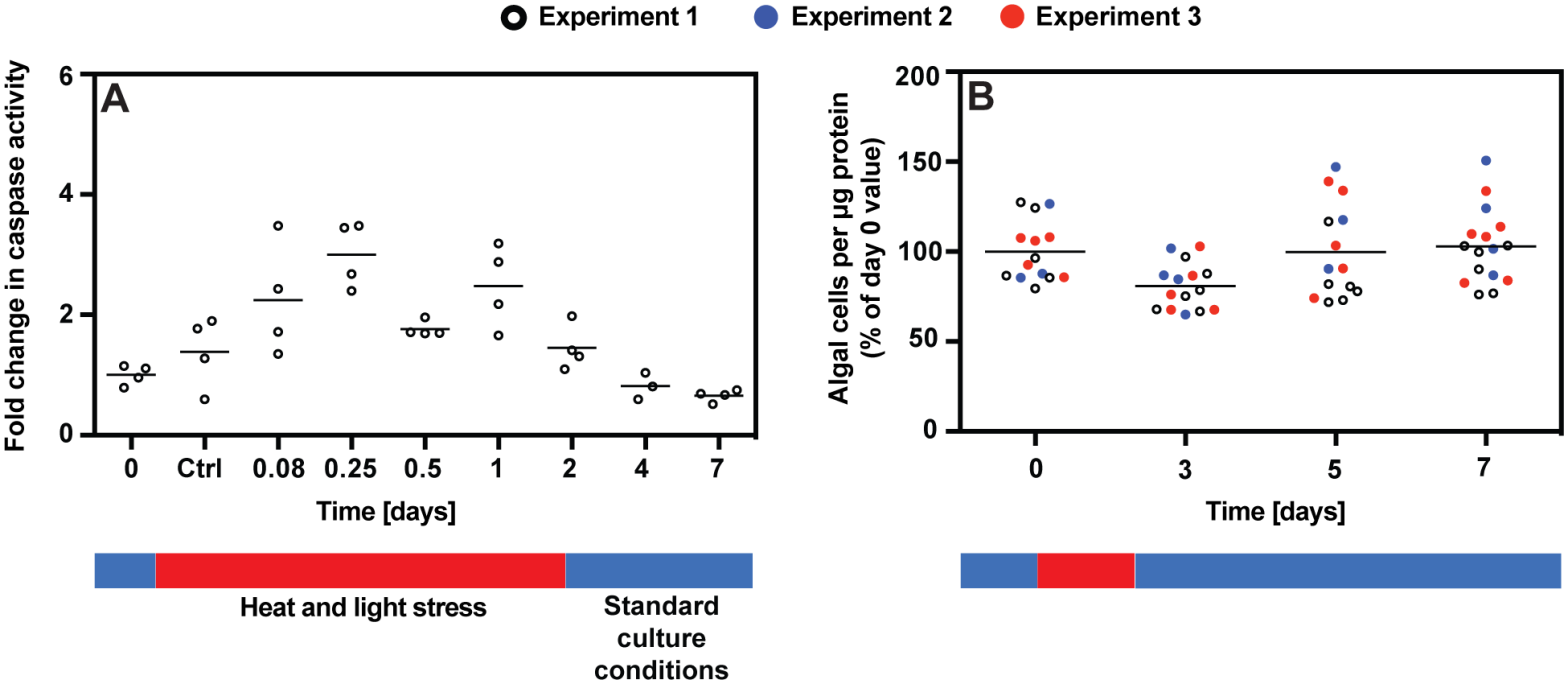
Lack of bleaching despite transient stimulation of apoptosis in *Aiptasia* exposed to limited-duration heat and light stress. Experiments were identical to those of Fig 2C and 2D except that after 2 d of stress, anemones were returned to standard culture conditions for the duration of the experiment. (A) Caspase activities were measured during a single experiment of this type. However, note that for the first 2 d, the experimental conditions were identical to those of the three replicate experiments of Fig 2C. (B) Algal numbers were measured during three replicate experiments of this type, one of which was the same as that used to obtain the caspase-activity values of panel A. In each panel, horizontal lines indicate the mean values for all animals at each time-point; none of these means appeared significantly different from the day-0 mean by the grouped ANOVA test.

**Fig 4:**
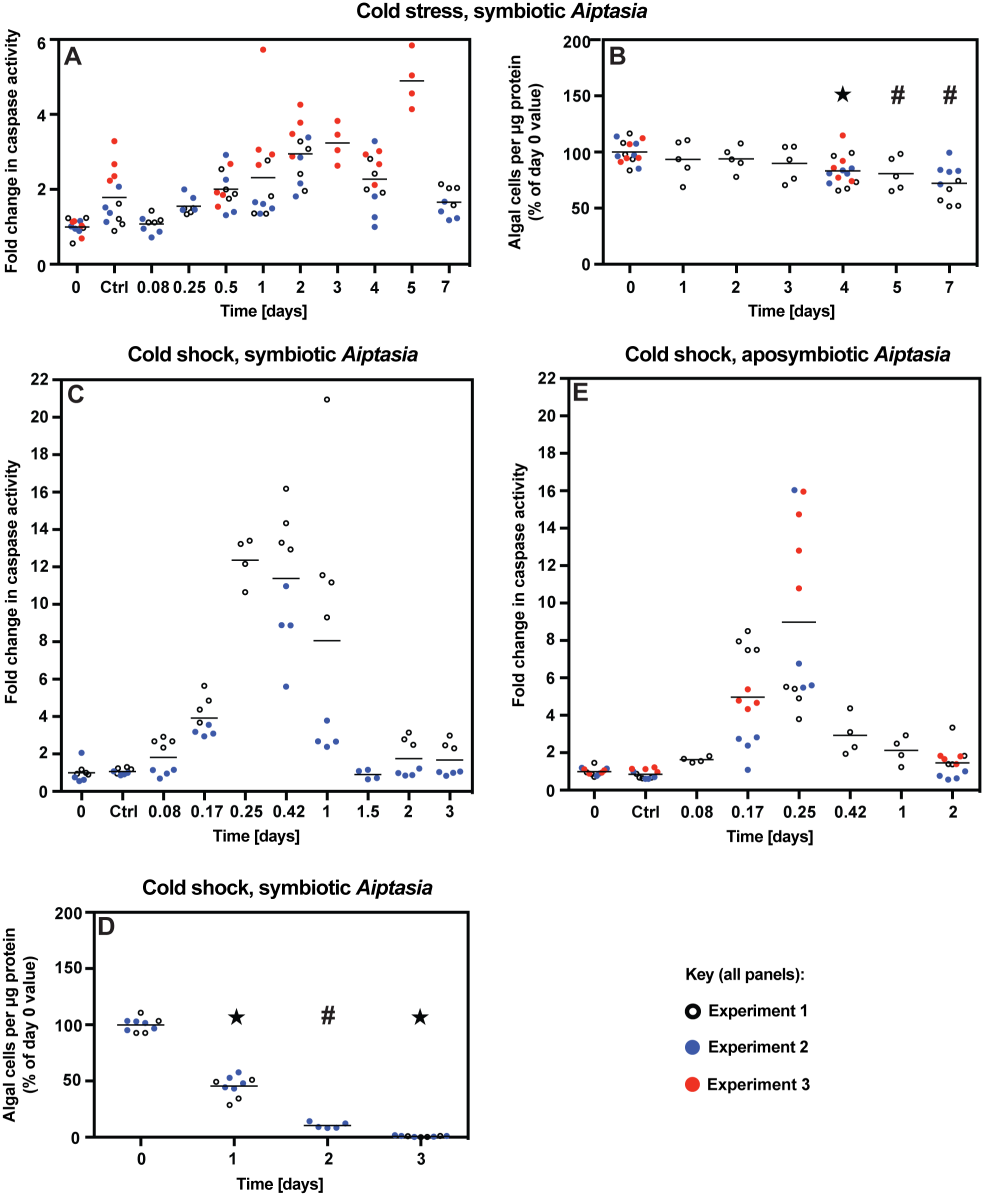
Imperfect correlation between the timing of apoptotic activity and that of bleaching under mild or acute cold stress. (A,B) Caspase activation and bleaching were assessed as in Fig 2 during mild cold stress (18°C, 25 μmol photons m^-2^ s^-1^); three independent experiments were performed. Note that the data for the 5-d time-point in A are from a single experiment and do not fit the overall trends; we presume that some unknown anomaly produced these outlier measurements. (C,D) Symbiotic anemones previously acclimated under standard culture conditions were shifted abruptly to 4°C in the dark, held for 4 h, and then returned to standard culture conditions for the duration of each experiment. Samples from two independent experiments were analyzed as in A,B. Note that the scale for C differs by a factor of two from that in the other panels showing caspase data. (E) Aposymbiotic animals were analyzed as in C; three independent experiments were performed. Scale as in panel C. In all panels, horizontal lines indicate the mean values for all animals at that time-point. In B and D, the results of grouped ANOVA and unpaired *t* tests are indicated as in Fig 2.

**Fig 5:**
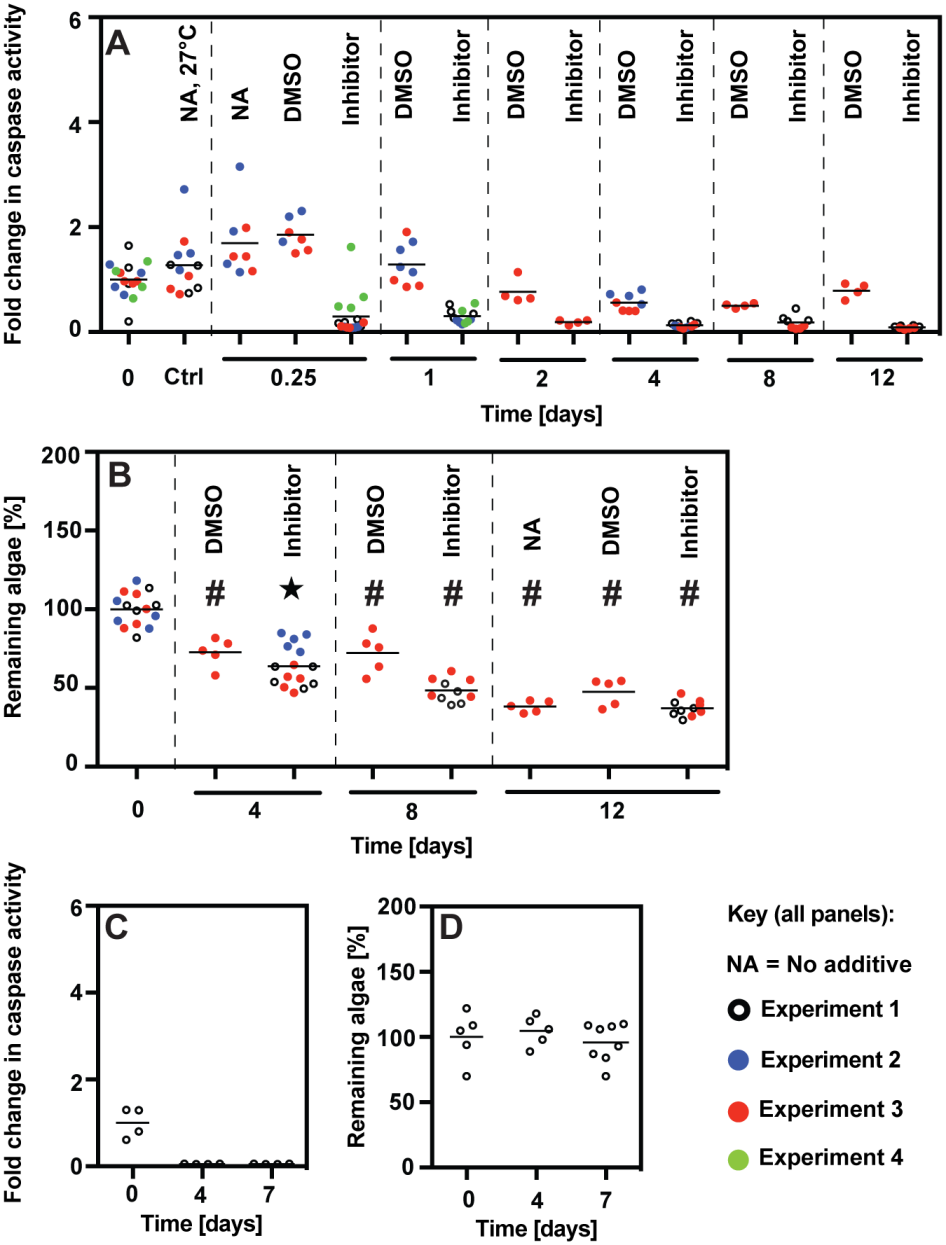
Occurrence of bleaching under heat and light stress despite inhibition of caspases (and hence presumably of apoptosis). (A,B) Four independent experiments were like those of Fig 2C and 2D except that some tubs were treated with the caspase inhibitor Ac-DEVD-CHO at 2 μM (added from a stock solution in DMSO) or with the same concentration of DMSO alone (see Materials and Methods). Experiment 4 was of only short duration and was intended only to provide additional evidence of the effectiveness of the caspase inhibitor during the time when caspase activities would otherwise be elevated; no algal counts were performed. (A) Caspase activities. As in Figs 2–4, the section labeled "Ctrl" shows measurements on anemones that were held under control conditions (27°C, no additives) for the full 12 (Experiments 1 and 3) or 4 (Experiment 2) days of the experiments. (B) Numbers of algae per unit protein. In each panel, horizontal lines indicate the mean values for all animals at that time-point. In B, the results of grouped ANOVA and unpaired *t* tests are indicated as in Fig 2. (C,D) In a single experiment, anemones under otherwise standard culture conditions (27°C, 25 μmol photons m^-2^ s^-1^) were treated with Ac-DEVD-CHO (2 μM final concentration) and monitored for caspase activities (C) and numbers of remaining algae (D).

**Fig 6:**
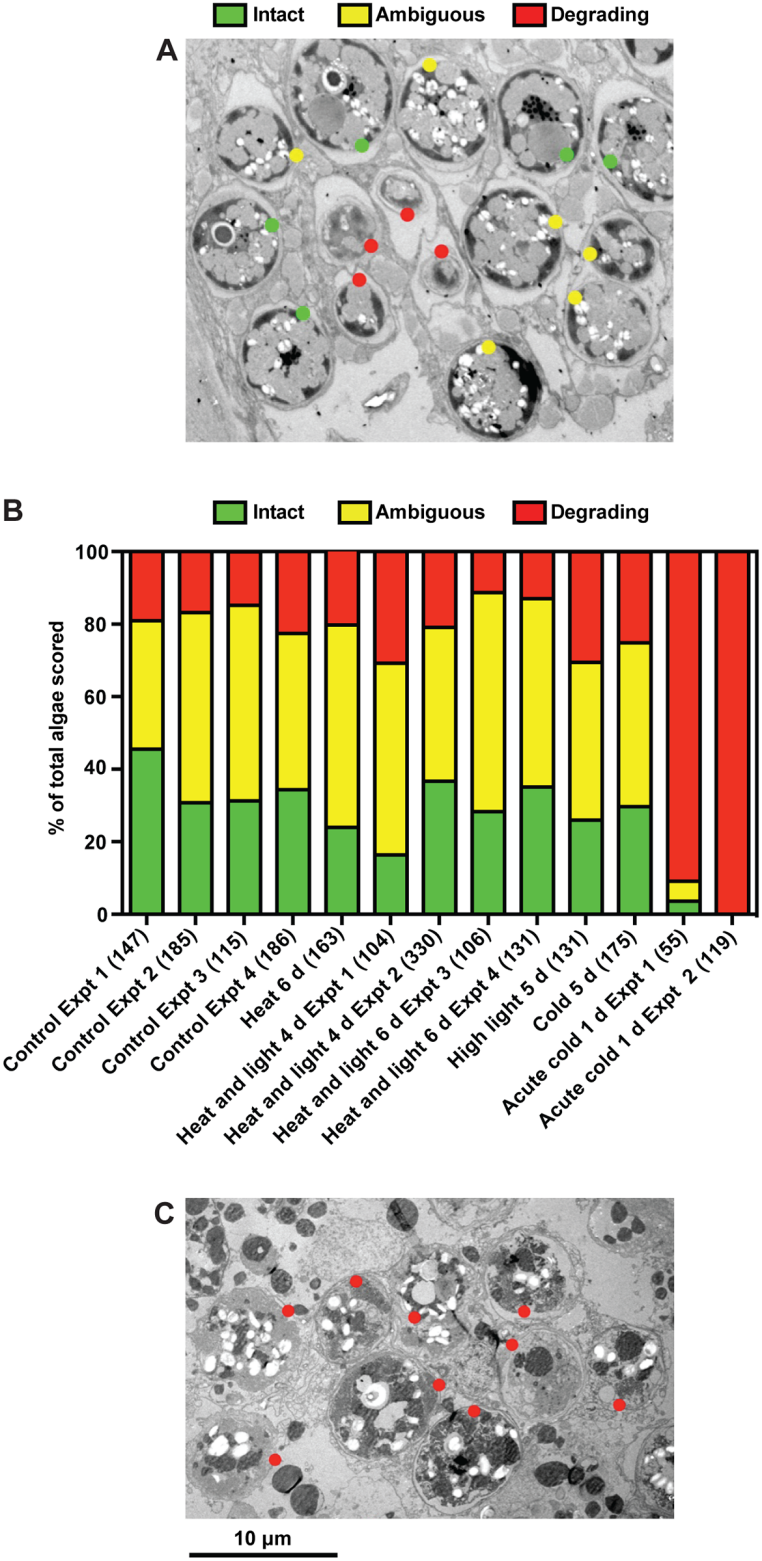
Increased *in situ* degradation of algae during acute cold shock but not under other stress conditions. In experiments like those of Figs 2A–2F and 4A–4D, symbiotic anemones were fixed after collection from populations under standard culture conditions (control experiments 1–4) or under stress conditions at times coinciding with maximal rates of bleaching (for acute cold shock, this is1 d after the return to standard culture conditions). Anemone sections were then examined by electron microscopy as described in Materials and Methods. In each experiment, every algal profile observed in multiple fields of a randomly chosen section of one animal was classified as Intact, Degrading, or Ambiguous following the examples shown in a section of an anemone from standard culture conditions (A; and see Materials and Methods). (B) Percentages of algae classified into each category in anemones exposed to the various stresses. Total numbers of algal profiles scored are indicated in parentheses. (C) A typical section from one of the acute-cold-shock experiments.

**Fig 7:**
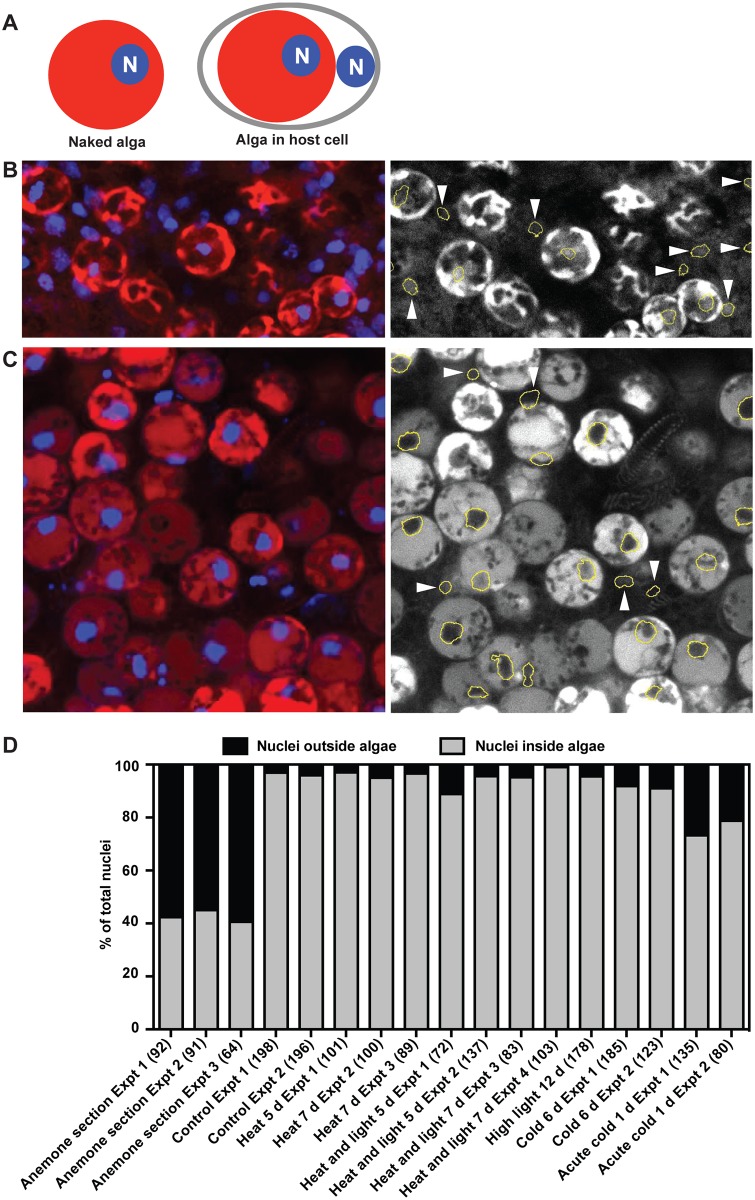
Apparent predominance of algal expulsion over host-cell detachment during bleaching under various stress conditions. In experiments like those of Figs 2A–2F and 4A–4D, samples of released material (typically small boluses) were collected from the ASW containing anemones under standard culture conditions and at times coinciding with significant rates of bleaching under stress (for acute cold shock, this is 1 d after the return to standard culture conditions). These samples were fixed, stained with DAPI, and examined by confocal microscopy as described in Materials and Methods. For each independent experiment, three separate samples of released material were examined in a *z*-stack of ~20 optical sections, and each nucleus was scored for whether it was inside an algal cell (an algal nucleus) or not (a host-cell nucleus), as judged by comparison to the chlorophyll fluorescence from the algal chloroplasts (which are largely concentrated in the cell cortex—see Figs 6A and 8C). The same scoring strategy was used with physical sections of intact anemones embedded as described in Materials and Methods. One of the three animals in which sections were examined had been grown under standard conditions; the other two had been subjected to heat and light stress for 6 and 48 h, respectively. (A) Diagram illustrating the scoring strategy. Expulsion of algae would yield "naked" algae in which the associated nuclei (N) would be seen to be within the algal cells (left), whereas host-cell detachment would reveal host-cell nuclei that were associated with, but not within, algal cells (right). (B and C) Single confocal optical sections of (B) a physical section of an intact anemone and (C) a sample of material released from animals subjected to acute cold shock, to illustrate the scoring diagrammed in A. The outlines of nuclei determined by image-analysis software (see Materials and Methods) were overlaid on the red-channel images of algal cells revealed by chlorophyll autofluorescence; arrowheads indicate nuclei that appear to be outside algae. (D) Percentages of nuclei scored as inside and outside algal cells in anemone sections and in the material released under different conditions; independent experiments are shown separately, and the total numbers of nuclei scored in each such experiment are indicated in parentheses.

Some of the data presented in this study (caspase measurements in Figs 2–5; quantitation of morphological observations in Figs 6 and 7) offered no clear path to informative statistical analyses but can be interpreted reliably without them. For statistical analyses of the bleaching data of Figs 2–5, the values (numbers of algae per unit protein) obtained for the individual anemones in a single tub were treated as replicate rather than independent measurements wherever possible. We took this conservative approach because although the results show that there is much more variation from animal to animal within a tub than can be attributed to the imprecision of the assays used (the algal counts and total-protein assays both have precision within a few per cent of the mean) [51], we also cannot be certain that there were not (unknown) features of their common environment that affected all animals in one tub in the same way. Independent experiments performed under the same conditions were then treated as separate groups, and the mean values over all groups for a given condition were compared using two-way ANOVA performed using the software GraphPad Prism with the null hypothesis of no bleaching. Mean values that appear to be significantly different (*P* ≤ 0.005) from the value at day 0 are indicated by a ★. In some cases of interest, this two-way ANOVA could not be applied because the numbers of groups differed between time-points; in these cases, unpaired *t* tests (in which the animals were treated as independent biological replicates) were performed. Mean values that appear to be significantly different (*P* ≤ 0.005) from the value at day 0 are indicated by a **#**.

## Results

### Evidence against an important role for apoptosis in bleaching

To monitor apoptotic activity under various stress conditions that lead to bleaching, we measured host caspase activities using an assay that should detect caspase-3-like and caspase-7-like enzymes (see Materials and Methods and S1 Fig), which are expected to play a central role in the execution phase of apoptosis as they are known to do in other animals [53]. In symbiotic anemones subjected to heat stress or a combined heat-and-light stress, there was an increase in caspase activity during the first ~1 d after the onset of stress conditions, after which the activity returned to baseline levels (Fig 2A and 2C). Because bleaching was not detectable until several days later and then continued for at least a week (Fig 2B and 2D; cf. also Fig 5B), it seems unlikely that the apparent early wave of apoptotic activity could be responsible for this bleaching. Moreover, high-light stress without heat stress caused bleaching of anemones without a detectable activation of caspase (Fig 2E and 2F), and a very similar transient increase in caspase activity was also observed in aposymbiotic animals exposed to heat-and-light stress (Fig 2G), indicating that the apparent transient apoptotic response to stress is independent of the presence of symbiotic algae.

Nonetheless, we also considered the possibility that a temporary activation of apoptotic pathways could lead to later bleaching. To test this possibility, anemones were exposed to heat-and-light stress for 2 d and then returned to standard culture conditions. As in the other heat-and-light-stress experiments, there was a transient activation of caspase during the first ~1 d (Fig 3A). Although the animals examined at 3 d may have been slightly bleached, the animals examined at 5 or 7 d showed no such effect (Fig 3B). These data support the conclusion that the apparent early wave of apoptosis was not responsible for the bleaching observed at later times in the experiments involving continuous heat-and-light stress (Fig 2C and 2D).

The results obtained with cold stress were somewhat less clear but still consistent with the hypothesis that apoptosis is not primarily responsible for bleaching. Under mild cold stress at 18°C, there was a slow rise in caspase activity over ~3 d followed by a slow return to baseline values (Fig 4A). In contrast, bleaching appeared to proceed slowly and steadily from ~3 d until the experiments were ended at 7 d (Fig 4B), so that the rate of bleaching was not well correlated with the apparent level of apoptotic activity. Similarly, although anemones exposed to acute cold shock exhibited an apparent burst of apoptotic activity over the first day that coincided with a rapid loss of algae during that time (Fig 4C and 4D), algal loss appeared to continue unabated for several days after caspase activities had returned to baseline values (Fig 4C and 4D). Given the drastic effects of 4°C shock on algal structure and function (see below), it seemed possible that the Guava counts overestimated the rate of algal loss by failing to count algae that remained in the host but had lost chlorophyll fluorescence. However, inspection of the actual Guava plots (S2 Fig) provided no support for this possibility. Finally, as with heat-and-light-stressed animals (see above), aposymbiotic animals subjected to acute cold shock displayed a peak in caspase activity that appeared indistinguishable from that observed in symbiotic animals (Fig 4E).

As a further test of the possible role of apoptosis in bleaching, we asked if inhibition of apoptosis via inhibition of caspases could prevent bleaching in anemones exposed to heat-and-light stress. Anemones were stressed as in the previous experiments but in the presence of Ac-DEVD-CHO, a highly effective inhibitor of mammalian caspases 3, 7, and 8 [49,50]. The inhibitor suppressed *Aiptasia* caspase activity effectively (Fig 5A), but bleaching proceeded at least as rapidly as in the controls (Fig 5B), providing strong support for the conclusion that apoptosis does not play a major role in bleaching under these conditions. A control experiment showed that the inhibitor itself did not cause bleaching in the absence of other stress (Fig 5C and 5D).

Finally, because we cannot be certain that assaying/inhibiting caspase(s) would detect/block all apoptosis-like activity, we also used electron microscopy to examine the host gastrodermal cells. In comparing sections of an anemone grown under standard culture conditions to sections of one undergoing bleaching after 6 d of heat-and-light stress (*cf*. Fig 2D), we could see no significant differences in the numbers, sizes, shapes, or general appearance of mitochondria, in the appearance and distribution of other intracellular membranes, or in the morphology of the mesoglea and the relationship of the gastrodermal cells to it (S3 Fig).

### Evidence against an important role for *in situ* degradation in bleaching under most stress conditions

To evaluate the possible role in bleaching of *in situ* degradation of algal cells, we examined anemone sections by electron microscopy after subjecting the animals to various stress conditions. Individual algal cells were scored as "intact", "degrading", or "ambiguous" (i.e., not clearly assignable to either the "intact" or "degrading" category) as shown in Fig 6A and described in Materials and Methods. Interestingly, nearly 20% of the algae were scored as degrading, with another ~45% scored as ambiguous, even in control animals (Fig 6B), consistent with some previous reports [22,54]. These percentages did not appear to change significantly in animals subjected to various stress conditions (Fig 6B) with the exception of acute cold shock, where nearly all algae could be scored unequivocally as degrading (Fig 6B and 6C). These data suggest that the rapid bleaching observed under acute cold shock (Fig 4D) is due, at least in part, to greatly accelerated rates of lysosomal, autophagic, apoptotic, or necrotic degradation of algae under these conditions but that such mechanisms play little or no role under other stress conditions.

However, the numbers of algae that could not be scored unambiguously as either intact or degrading were substantial under all conditions (except acute cold shock), and we have no reliable estimate of the kinetics of the degradation process. Thus, it seemed conceivable that a small increase in the fraction of algae scored as degrading (or possibly so) in a fixed sample might reflect a large increase in the actual numbers of algae being degraded per unit time. To address this possibility, we estimated the numbers of algae to be accounted for in animals subjected to 7 d of heat-and-light stress [those estimated to have been present initially (Table 2, Row 3) plus those estimated to have been generated by algal reproduction during the experiment (Table 2, Row 5)] and compared them to the numbers of algae whose fate was known [those remaining in the stressed animals (Table 2, Row 7) plus those collected from the surrounding medium during the experiment (Table 2, Row 8)]. In each of three independent experiments, it appeared that there were no "missing" algae to be accounted for by complete degradation *in situ* (Table 2, compare Rows 6 and 9). Of the several assumptions needed for these calculations, the two most problematic appear to be (i) that the relative biomasses of the stressed and control anemones when they were measured at 7 d were the same as they had been at 0 d (it is likely that the stressed anemones had lost more total biomass over the 7 d) and (ii) that we were able to achieve collection of 100% of the shed algae from the surrounding ASW (it is likely that some algae were missed—despite the small-volume wells used for these experiments—because they were stuck to the walls of the chambers or to the anemones themselves). As these two probable errors would affect the comparison of interest in opposite directions, our conclusion that few, if any, algae were completely degraded *in situ* during bleaching under heat-and-light stress seems likely to be correct.

**Table 2:** Evidence against a quantitatively significant role for complete degradation *in situ* during bleaching of *Aiptasia* under heat-and-light stress^a^.

		Experiment		
Row	Parameter ^b^	1	2	3
1	Total algae in control anemones at 7 d	5.2	2.4	5.7
2	Total protein in stressed anemones ÷ total protein in control anemones (both values from 7 d)	0.63	1.0	0.81
3	Estimated total algae in stressed anemones at 0 d (Row 1 x Row 2) ^c^	3.3	2.4	4.6
4	Total algae released by control anemones in 7 d	1.8	0.24	0.45
5	Estimated number of algae produced by reproduction in stressed anemones over 7 d (Row 4 x Row 2) ^d^	1.1	0.24	0.36
6	Total algae from stressed anemones to be accounted for (Row 3 + Row 5)	4.4	2.6	5.0
7	Total algae remaining in stressed anemones at 7 d	1.4	2.0	2.9
8	Total algae released by stressed anemones in 7 d	3.3	0.62 ^e^	2.9
9	Total algae accounted for in stressed anemones (Row 7 + Row 8; to be compared to Row 6)	4.7	2.6 ^e^	5.8

^a^ Each experiment used 10 anemones from a single tub that had been incubated under standard growth conditions (27°C, 25 μM photons m^-2^ s^-1^) for at least 21 days to acclimate the anemones. The 10 anemones were selected to be approximately the same size as judged by eye. In each experiment, five of the anemones were placed in ~10 ml of ASW in one well of a 6-well plate that was then incubated under stress conditions (34°C, 150 μM photons m^-2^ s^-1^) for 7 d with daily water changes. All stressed animals in all three experiments survived and indeed seemed to be grossly normal in behavior and in tissue architecture (S3 Fig), except for the losses of algae. In parallel, the remaining five anemones (control) were incubated in one well of another plate under standard growth conditions for 7 d. At each water change, the used ASW was centrifuged, and the pellet was resuspended in 1.5 ml (Experiment 1) or 1.25 ml (Experiments 2 and 3) of MilliQ water containing 0.01% SDS and frozen; these samples were analyzed by Guava flow cytometry to provide the measured data included in rows 4 and 8. At 7 d, anemones were homogenized individually, and their total numbers of algae and total protein contents were determined by Guava flow cytometry and BCA assay, respectively. These amounts were then summed for each group of five anemones to provide the measured data included in rows 1, 2, and 7.

^b^ Except for the ratio in Row 2, all values are in millions of algae.

^c^ We assume that the relative protein contents of the two sets of anemones at 7 d reflect their relative biomasses at 0 d.

^d^ We make three assumptions. (1) There is negligible reproduction of the released algae in the ASW prior to their collection during water changes. This assumption is supported by the low rate of reproduction (doubling time of ≥10 d) of *Symbiodinium* strain SSA01 in pure culture (our unpublished results; see also Xiang *et al*. 2013). (2) The control anemones are in steady state, so that the number of algae collected in the water changes equals the net reproduction of algae within the host during the 7 d incubation. This assumption seems reasonable given (i) the long periods of acclimation under identical conditions before the start of each 7-day experimental period and (ii) the lack of feeding during the experimental period, resulting in limited (if any) growth of the animals during that period. (3) The net rate of algal reproduction in the host is similar (on a unit-biomass basis) under the control and stress conditions. This assumption is difficult to test, but we note that at least the ratio of intact to degrading algae *in situ* appears to be similar in the two cases (Fig 6). Moreover, if this assumption were incorrect, and the rate of algal reproduction were less under the stress conclusions, the effect would be to decrease the values estimated in Rows 5 and 6 and thus to strengthen, rather than weaken, the conclusion that all algae lost have been accounted for by those found in the water.

^e^ It is likely that the percentage of algae that were not successfully collected and counted was highest in this case with the lowest absolute number of algae.

### Evidence that expulsion of algae, but not host-cell detachment, plays an important role in bleaching

The algae expelled into the surrounding medium under stress (Table 2) might be either "naked", implying release by exocytosis or a related mechanism, or still present in host cells, implying an exfoliation of gastrodermal cells containing algae. To evaluate these possibilities, we collected the material released into the ASW around the anemones, stained nuclei with DAPI, and examined the cells by confocal microscopy, reasoning that naked algae would always show one or two nuclei within the boundaries of the algal cell (marked by its red chlorophyll fluorescence), whereas exfoliated gastrodermal cells would often show the host-cell nucleus associated with, but clearly external to, the algal cell (Fig 7A). Individual nuclei were followed through *z*-stacks of confocal images and scored manually as either being inside or outside of algal cells. Both control experiments with sections of intact anemones (Fig 7B and 7D) and observations on material released from anemones subjected to acute cold shock (where some significant exfoliation of gastrodermal cells seems to occur: Fig 7C and 7D) allowed us to verify that we could indeed recognize host nuclei in this way. The observations on the cells released after acute cold shock also indicated that exfoliated host cells did not invariably disintegrate too rapidly to be captured by our methods. Nonetheless, we found that under standard growth conditions and all the stress conditions tested other than acute cold shock, essentially all released material was in the form of naked algae (Fig 7D). Thus, it appears that with the partial exception of acute cold shock, expulsion of algae by exocytosis or a related mechanism plays by far the most important role, with exfoliation of algae-containing gastrodermal cells contributing very little to the bleaching process.

### Severe damage to algae during acute cold shock

**Fig 8:**
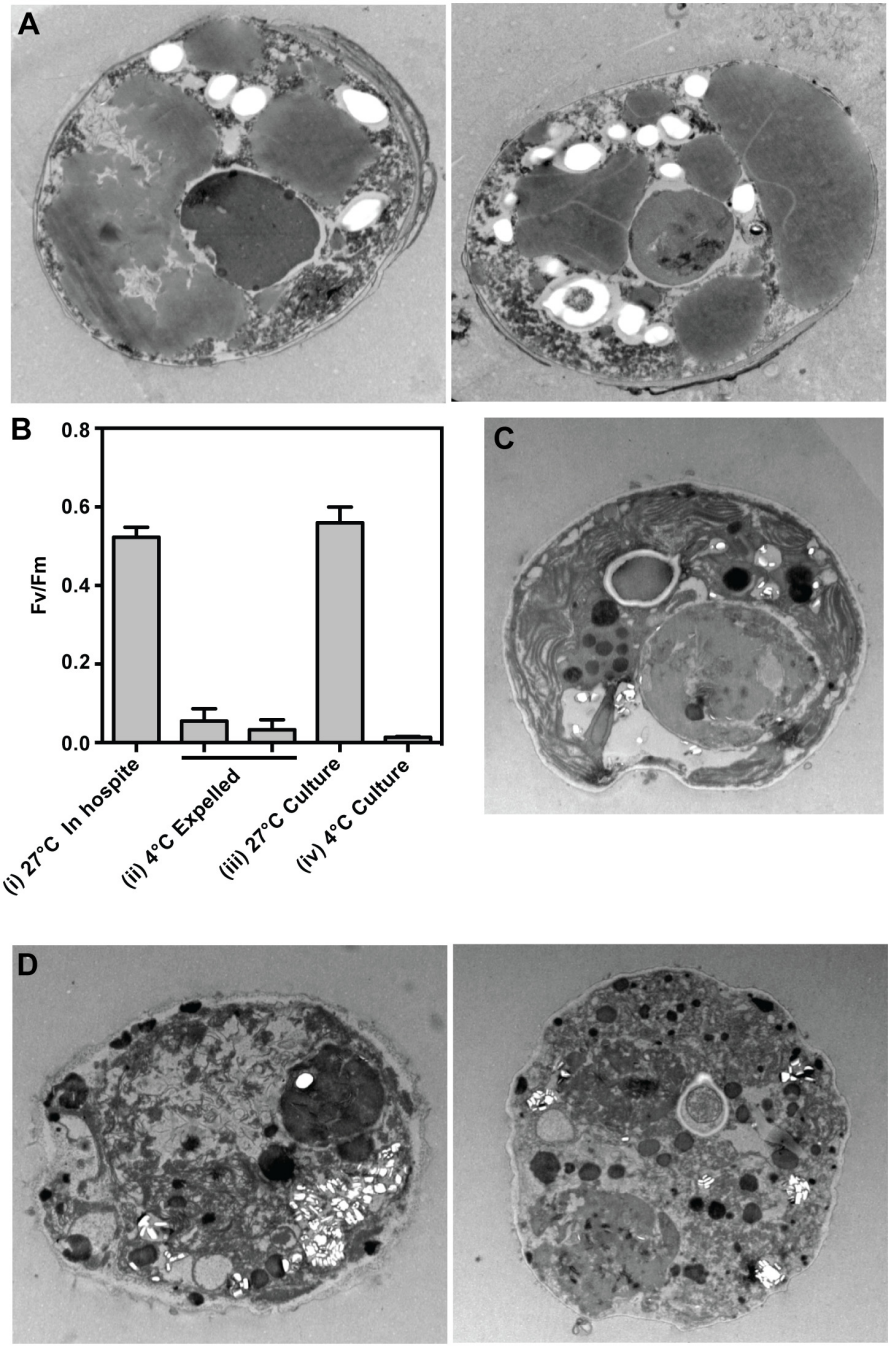
Severe damage to algae exposed to acute cold shock either *in hospite* or in culture. (A) In an experiment like that of Fig 4C and 4D, algae collected from the ASW 24 h after the end of the cold shock and return of the anemones to standard culture conditions were fixed and examined by electron microscopy; algae *in hospite* in unperturbed animals (Fig 6A) provide a comparison. (B) Maximum quantum yields of Photosystem II as judged by *F*_*v*_*/F*_*m*_ for (i) algae *in hospite* in anemones under standard culture conditions (n = 3 animals; mean ± SD is shown); (ii) algae collected from the ASW in tubs that contained ~45 (left) or ~75 (right) anemones 24 h after an acute cold shock to the anemones [as in Fig 4C and 4D; means ± SDs of two (left) or three (right) technical replicates are shown]; (iii) cultured algae (strain SSA01) growing at 27°C, 10 μmol photons m^-2^ s^-1^ (three replicate experiments were performed using separately grown cultures; mean ± SD is shown); and (iv) cultured algae that were collected 24 h after an acute cold shock as in Fig 4C and 4D but with the incubation after the shock at 27°C, 10 μmol photons m^-2^ s^-1^ (three replicate experiments were performed using separately grown cultures; mean ± SD is shown). (C,D) Cultured algae of strain SSA01 were fixed and examined by electron microscopy (C) during growth at 27°C, 10 μmol photons m^-2^ s^-1^ or (D) 24 h after a 4-h cold shock in the dark as in Fig 4C and 4D but with the incubation after the shock at 27°C, 10 μmol photons m^-2^ s^-1^.

The effects of acute cold shock were so strikingly (and surprisingly) different from those observed under other conditions that we explored these effects further. Given the apparent degradation of the algae even *in hospite* (Fig 6B and 6C), it was not surprising that the algae released from the host (and collected from the surrounding ASW) also appeared badly degraded, with little trace either of normal intracellular structure (Fig 8A) or of normal Photosystem II function (Fig 8Bi and 8Bii). To ask if the deterioration of the algae under cold shock is an intrinsic property of the algae or results from damage inflicted by a stressed host, we performed similar experiments with cultured algae of strain SSA01, which was derived from the anemones used in the other experiments (see Materials and Methods). At 27°C, cultured algae showed internal structure similar to that of healthy algae *in hospite* at the same temperature (Fig 8C; *cf*. the "intact" algae in Fig 6A), but cultured algae that had been subjected to cold shock showed a loss of both normal internal structure (Fig 8D) and Photosystem II function (Fig 8Biii and 8Biv).

## Discussion

Multiple possible cellular mechanisms of algal loss during cnidarian bleaching have been proposed and indeed reported in previous studies (see Introduction and Fig 1). However, it appears that no previous study has attempted a quantitative comparison of the relative contributions of the different possible mechanisms to the bleaching of a single type of organism under various stress conditions. In this study, we evaluated the possible mechanisms in parallel in individuals from a clonal population of the sea anemone *Aiptasia* that were exposed to precisely controlled stress conditions of heat alone, high light alone, heat and moderately high light, or different degrees of cold. Our results indicate that under all conditions tested except acute cold shock, host-cell (or algal) apoptosis, *in situ* degradation of algae, and detachment of gastrodermal cells containing algae into the environment all play quantitatively negligible roles in algal loss, which appears to be accounted for almost entirely by expulsion of algae by exocytosis or a related mechanism. Interestingly, under acute cold shock at 4°C, both *in situ* degradation and gastrodermal-cell release appear to become significant, although even under these conditions, expulsion of the (mostly degraded) algae appears to be the predominant mechanism of algal loss.

The many differences between our study and previous ones make it difficult to explain the largely discrepant results. Perhaps most puzzling is our different assessment of the possible role of apoptosis, particularly as several of the previous studies have also employed *Aiptasia* and stress conditions similar to those used here [13,14,26,28]. However, we feel that the case against an important role for apoptosis in bleaching in our experiments is strong. Examination of the *Aiptasia* genome and transcriptome revealed at least one gene, AIPGENE14128, whose product seems likely to play an "executioner" role in *Aiptasia* apoptosis based on its strong sequence similarity to human caspases 3 and 7 (S1 Fig). Similarly, the peptides used to assay and inhibit caspase in our experiments would be expected to be effective with the 14128 gene product [49,50,55], and possibly also with other caspase-like proteins (S1 Fig). Using the peptide assay, although we found that most stresses produced a transient peak of caspase activity (presumably signaling a transient wave of apoptosis), there was no correlation between the timing of this peak and the timing of bleaching, with the partial (and unconvincing) exception of cold stress. Moreover, (i) electron microscopy of anemone sections at the time when bleaching was occurring showed no obvious differences in host-cell morphology; (ii) the early peak of (presumed) apoptosis under heat-and-light stress was not sufficient to produce later bleaching when this possibility was tested directly; (iii) stress by high light without heat produced bleaching without any detectable peak of caspase activity; (iv) although the caspase inhibitor was effective in suppressing caspase activity, it had no detectable effect on bleaching; and (v) aposymbiotic anemones displayed a very similar peak of caspase activity under stress, suggesting that the apoptotic activity is a generalized short-term response of the animal cells to the stress rather than a specific response to the presence of damaged algal cells. In this regard, it may also be relevant that some earlier studies reported that reactive-oxygen species, one potential trigger for apoptotic pathways [6], are produced under thermal stress in an algae-independent manner [56,57]. Finally, although it is conceivable that host-cell apoptosis could result in the release of intact algae into the medium, it seems more likely that the algae would also be destroyed; thus, our apparent ability to account for all of the algae lost by the host by quantifying the algae collected from the medium (Table 2) becomes another argument against a quantitatively significant role for apoptosis in bleaching.

Testing the possible role of *in situ* degradation of algae is challenging because even under control conditions, many algae appear to be undergoing degradation at any given time, as judged by electron microscopy (Fig 6A and 6B), as noted also by others [22,54]. Except in the case of cold shock (see below), we detected no significant increase in the fraction of algae that appeared to be degrading *in situ* during stress, but a small change in this fraction could represent a large increase in the numbers of algae degraded if the process were rapid (which we consider unlikely but cannot rule out). However, when we compared the numbers of algae lost from the hosts to the numbers collected from the surrounding medium during heat-and-light stress, we found no significant difference (Table 2), which appears to rule out a quantitatively significant contribution of complete degradation of algae *in situ* to algal loss from the host under these conditions.

When we used nuclear staining and confocal microscopy to assess the relative contributions of expulsion of algal cells and host-cell detachment, we observed almost exclusively what appeared to be free algal cells (except in the case of cold shock—see below), suggesting that release of algae by exocytosis or a related mechanism was by far the predominant mode of algal loss. This conclusion is also supported by a failure to observe signs of host-cell detachment in electron micrographs of sections of anemones bleaching in response to heat-and-light stress either in our study or in that of Hanes and Kempf [21]. In contrast, some earlier studies suggested a significant role for host-cell detachment [11,24]. In particular, Gates et al. [24] used both light and electron microscopy to provide clear evidence that host-cell detachment and release to the environment could occur. However, this study provided no quantitation of such detachment in comparison to possible expulsion of algal cells, and all of the data shown were from anemones subjected to cold-shock, a condition under which we also observed a significant number of seemingly detached and released host cells. This last observation provides some evidence against, but does not totally rule out, a possibility suggested by Gates et al. [24], namely that significant numbers of host cells are detached as such but then quickly break down in the environment to release the algae. Similarly, because we used only nuclear staining to evaluate whether the released algae were still contained within host cells, we cannot say whether the algae released are truly "naked" or still encased in the host-derived symbiosome membrane and/or portions of plasma membrane as in the apocrine-like release mechanism reported by Hanes and Kempf [21]. Resolution of these issues will require further studies by both electron microscopy and live-cell microscopy of bleaching animals.

Surprisingly, cold shock at 4°C elicited bleaching mechanisms that appeared to have little or no role under the other stress conditions tested in our study. In particular, the numbers of detached host cells increased significantly, and the degradation of algae *in situ* increased dramatically. The degraded algae were also released from the host, but appeared severely damaged when collected from the medium. The sensitivity to cold shock appeared to be an intrinsic property of the algae rather than a product of damage inflicted by the stressed host, as very similar degradation was seen when cultured algae were subjected to a 4°C shock, whereas the hosts uniformly survive a 4°C cold shock without obvious detriment other than the loss of algae. Although loss of photosynthetic activity by algae subjected to cold shock has also been observed previously [23], the severe effects of the cold shock on the algae still seemed surprising given the ability of many mesophilic prokaryotic and eukaryotic microbes to robustly withstand exposure to 4°C. These severe effects of cold shock presumably reflect a lack of selection in these tropical organisms for mechanisms to deal with a temperature that they would very rarely encounter in nature, but a mechanistic understanding must await future investigation. An interesting possibility is that the increase in host-cell detachment is a secondary response by the host to the presence of a large population of badly damaged algae.

In summary, our study suggests that under most stress conditions—and, in particular, under the ecologically relevant combination of heat and light stress—expulsion of algae, by either an exocrine or apocrine [21] mechanism, is by far the predominant mechanism by which algae are lost in bleaching cnidarians. This conclusion is consistent with some [11,18–21,58], but not all, previous reports. It remains possible that different mechanisms predominate in other organisms or under stress conditions not tested here. However, validating such a conclusion will require a comparative and quantitative evaluation of the contributions by the various possible mechanisms to bleaching in genetically identical individuals exposed to different conditions. In any case, it remains the case that the molecular mechanisms that trigger algal release remain unknown, and it is not even clear whether it is the host or the alga that initiates the bleaching process. Gaining a deeper understanding of the molecular and cellular mechanisms of cnidarian bleaching is critical for monitoring the health and promoting the survival of corals and other cnidarians. Further studies in the experimentally tractable *Aiptasia* model system should facilitate efforts to this end.

## Supporting Information

S1 Fig Putative *Aiptasia* caspases. We used BLASTP to search the 29,269 gene models in the *Aiptasia* genome assembly [41] with human caspase 3 (GenBank NP_004337.2), caspase 7 (GenBank AAH15799.1), and caspase 9 (GenBank BAA82697.1) as the query sequences. With caspases 3 and 7, the top five hits were AIPGENEs 14128, 23203, 25497, 10739, and 23198, in that order. With caspase 9, the top five hits were the same, but in the order 23203, 25497, 10739, 14128, and 23198. Similarly, we searched the ~27,000 transcripts in the independently assembled *Aiptasia* transcriptome [36] using the same three query sequences. The top six hits (transcripts 63620_1, 106327_1, 54098_1, 54104_1, 90433_1, and 51289_1) were the same in each case (although the order varied), except that 51289_1 (the sixth best hit with caspase 3 and the fifth best hit with caspase 9) was the seventh best hit with caspase 7, with transcript 33459_1 appearing as the sixth best hit. The gene-model and transcript searches agreed well with each other. First, for three of the five top gene-model hits, there was one of the six (or seven) top transcript hits whose predicted amino-acid sequence matched perfectly (gene 14128, transcript 63620_1; gene 23203, transcript 106327_1; gene 10739, transcript 90433_1). The predicted product of gene 14128 also matches almost perfectly (just two amino-acid differences) the protein described previously as “acasp” (GenBank ABA62018.1) [59]. Second, the predicted products of gene 23198 and transcript 51289_1 matched perfectly except for 11 positions (of ~463); we have not explored the possible reasons for these differences. Finally, the predicted product of gene 25497 matches perfectly the predicted products of both transcripts 54098_1 and 54104_1 except that (i) although both transcripts predict the same C-terminus, both also lack a stretch of nine amino acids lying just nine amino acids upstream of this C-terminus in the predicted product of the gene model; (ii) the predicted product of the gene model continues for 393 amino acids beyond the C-terminus predicted by both transcripts; and (iii) transcripts 54098_1 and 54104_1 predict proteins that are 24 amino acids longer and 27 amino acids shorter, respectively, at the N-terminus than that predicted by the gene model. The issues at the C-terminus probably reflect an error (fusion of two genes) in the genome assembly, and we have not explored the possible reasons (alternative transcripts, assembly errors, etc.) for the discrepancies at the N-terminus. None of these gene-transcript discrepancies affects the comparisons to caspase sequences. (A-C) Comparison of the human caspase 3, 7, and 9 amino-acid sequences to those predicted for genes 14128 (A), 23203 (B), and 25497 (C). Each alignment runs to the C-terminus of the human protein. Residues involved in substrate recognition and catalysis are shown in red; yellow shading indicates the catalytic cysteine [55]. Note that the gene 14128 product resembles caspases 3 and 7 distinctly more than caspase 9, whereas assignments of specific caspase types to the products of genes 25497 and 23203 are less clear. This ambiguity also applies to the products of genes 10739, 23198, and 1063 (which aligns perfectly to that of transcript 33459_1; see above): in alignments like those in A-C, they showed per cent identities ranging from 27.0 (gene 1063 vs. caspase 9) to 35.4 (gene 10739 vs. caspase 3).
(PDF)

S2 Fig The raw Guava output for experiment 2 of Fig 4C and 4D. Red chlorophyll fluorescence is plotted against side scatter (a measure of particle size and refractive index) for particles passing through the laser beam in the flow cytometer. The red square denotes the window in which *Symbiodinium* cells normally fall. Additional details are provided elsewhere [51].
(PDF)

S3 Fig Maintenance of integrity by gastrodermal cells of anemones exposed to heat and light stress. In an experiment like that of Fig 2C and 2D, symbiotic anemones were fixed after collection from populations under standard culture conditions (A,B) or after 6 d of heat-and-light stress (C-E). Sections were then examined by electron microscopy as described in Materials and Methods. A and B are sections of different anemones; C-E are different fields of view in the same anemone section. A, alga; E, epidermis; G, gastrodermis; GC, gastric cavity; M, mitochondria; Mg, mesoglea. Scale bar (all panels), 10 μm. In E, conspicuous white line is a sectioning artifact, and the arrowhead indicates what may be either a detached host cell in the gastric cavity or an attached cell seen in this section because of distortion during embedding and sectioning. Note also seemingly free algal cells in the gastric cavity.
(PDF)

S4 Fig Tests for the presence of bacterial, fungal, or other contaminants in cultures of *Symbiodinium* strain SSA01. (A) SSA01 cells (see Materials and Methods) were grown in liquid IMK medium containing both casein hydrolysate and glucose (so that heterotrophic contaminants could grow), stained with DAPI, and examined by DIC and fluorescence microscopy as described in Materials and Methods. Scale bar, 5 μm. (B) DNA from the cultured cells was used as template for attempted amplification of bacterial 16*S* rDNA by PCR (see Materials and Methods). Amplification of the 16*S* fragment from *E*. *coli* (using the same primers and conditions) and of the *Symbiodinium* rDNA ITS2 region served as controls.
(PDF)

## Abbreviations

Ac-DEVD-AFC: N-Acetyl-Asp-Glu-Val-Asp-7-amino-4-trifluoromethylcoumarin
Ac-DEVD-CHO: N-Acetyl-Asp-Glu-Val-Asp-aldehyde
ASW: artificial seawater
DAPI: 4',6-diamidino-2-phenylindole
DMSO: dimethyl sulfoxide
PBS: phosphate buffered saline
