# Supplementary material for: Relative Contributions of Various Cellular Mechanisms to Loss of Algae during Cnidarian Bleaching
Source: PLoS One. 2016 Apr 27;11(4):e0152693. doi: 10.1371/journal.pone.0152693 (PMC4847765; doi:10.1371/journal.pone.0152693)
Supplement: S1 Fig — We used BLASTP to search the 29,269 gene models in the Aiptasia genome assembly [41] with human caspase 3 (GenBank NP_004337.2), caspase 7 (GenBank AAH15799.1), and caspase 9 (GenBank BAA82697.1) as the query sequences. With caspases 3 and 7, the top five hits were AIPGENEs 14128, 23203, 25497, 10739, and 23198, in that order. With caspase 9, the top five hits were the same, but in the order 23203, 25497, 10739, 14128, and 23198. Similarly, we searched the ~27,000 transcripts in the independently assembled Aiptasia transcriptome [36] using the same three query sequences. The top six hits (transcripts 63620_1, 106327_1, 54098_1, 54104_1, 90433_1, and 51289_1) were the same in each case (although the order varied), except that 51289_1 (the sixth best hit with caspase 3 and the fifth best hit with caspase 9) was the seventh best hit with caspase 7, with transcript 33459_1 appearing as the sixth best hit. The gene-model and transcript searches agreed well with each other. First, for three of the five top gene-model hits, there was one of the six (or seven) top transcript hits whose predicted amino-acid sequence matched perfectly (gene 14128, transcript 63620_1; gene 23203, transcript 106327_1; gene 10739, transcript 90433_1). The predicted product of gene 14128 also matches almost perfectly (just two amino-acid differences) the protein described previously as “acasp” (GenBank ABA62018.1) [59]. Second, the predicted products of gene 23198 and transcript 51289_1 matched perfectly except for 11 positions (of ~463); we have not explored the possible reasons for these differences. Finally, the predicted product of gene 25497 matches perfectly the predicted products of both transcripts 54098_1 and 54104_1 except that (i) although both transcripts predict the same C-terminus, both also lack a stretch of nine amino acids lying just nine amino acids upstream of this C-terminus in the predicted product of the gene model; (ii) the predicted product of the gene model continues for [file pone.0152693.s001.pdf]

A GENE14128 (Transcript 63620_1), 416 amino acids

Fig. S1

Versus Human Caspase 3: 45% Identity over 238 amino acids

```
14128 170 YKMDKTPRGVAVIINNKSFLPASGMHRYPRNGTDVDRDALDKVFQKLGFNTLVYNDQSVYEIQKIFKSLAARDYSKENALIVSILTHGEE 259
Casp3  37 YKMDYPEMGLCIIINNKNFHKSTGMT--SRSGTDVDAANLRETFRNLKYEVRNKNDLTREEIVELMRDVSKEDHSKRSSFVCVLLSHGEE 124
          **** .  *:.:*****.*  ::**   .*.*****   * :.*::* ::.   ** :  ** ::::.:: .*:**..:::  :*:****

14128 260 GILYATDGTIQIRDMMRWFKG---TNLVGKPKIFIFQACQGHEYMDGVDATDAPPADKRVQ-IPVEADFLYAYSTVPGYYSWRNSVNGSW 345
Casp3 125 GIIFGTNGPVDLKKITNFFRGDRCRSLTGKPKLFIIQACRGTELDCGIETDSGVDDDMACHKIPVEADFLYAYSTAPGYYSWRNSKDGSW 214
          **::.*:*.::::.: .:*:*    .*.****:**:***:* *   *::: ..   *   : ************.********* :***

14128 346 FIQSIAEVFDKYAKTTDLLTMMTRVNALVAT-YQSRTNDPYSDRKKQIPSIVSMMRKDFYFYP  407
Casp3 215 FIQSLCAMLKQYADKLEFMHILTRVNRKVATEFESFSFDATFHAKKQIPCIVSMLTKELYFYH  277
          ****:. ::.:**.. ::: ::****  *** ::* : *.  . *****.****: *::***
```

Versus Human Caspase 7: 45% Identity over 238 amino acids

```
14128 170 YKMDKTPRGVAVIINNKSFLPASGMHRYPRNGTDVDRDALDKVFQKLGFNTLVYNDQSVYEIQKIFKSLAARDYSKENALIVSILTHGEE 259
Casp7  60 YNMNFEKLGKCIIINNKNFDKVTGMG--VRNGTDKDAEALFKCFRSLGFDVIVYNDCSCAKMQDLLKKASEEDHTNAACFACILLSHGEE 147
          *:*:    * .:*****.*  .:**    ***** * :** * *:.***:.:**** *  ::*.::*. : .*:::  .:   :*:****

14128 260 GILYATDGTIQIRDMMRWFKG---TNLVGKPKIFIFQACQGHEYMDGVDATDAPP----ADKRVQIPVEADFLYAYSTVPGYYSWRNSVN 342
Casp7 148 NVIYGKDGVTPIKDLTAHFRGDRCKTLLEKPKLFFIQACRGTELDDGIQADSGPINDTDANPRYKIPVEADFLFAYSTVPGYYSWRSPGR 237
          .::*..**.  *:*:   *:*   ..*: ***:*::***:* *  **::* ..*     *: * :********:***********.. .

14128 343 GSWFIQSIAEVFDKYAKTTDLLTMMTRVNALVA-TYQSRTNDPYSDRKKQIPSIVSMMRKDFYFYP 407
Casp7 238 GSWFVQALCSILEEHGKDLEIMQILTRVNDRVARHFESQSDDPHFHEKKQIPCVVSMLTKELYFSQ 303
          ****:*::..:::::.*  ::: ::****  **  ::*:::**: ..*****.:***: *::**
```

Versus Human Caspase 9: 31% Identity over 239 amino acids

```
14128 170 YKMDKTPRGVAVIINNKSFLPASGMHRYPRNGTDVDRDALDKVFQKLGFNTLVYNDQSVYEIQKIFKSLAARDYSKENALIVSILTHGEE 259
Casp9 153 YILSMEPCGHCLIINNVNFCRESGLR--TRTGSNIDCEKLRRRFSSPHFMVEVKGDLTAKKMVLALLELAQQDHGALDCCVVVILSHGCQ 240
          * :.  * * .:**** .*   **::  .*.*:::* : * : *..  * . * .* :. ::   : .** :*:.  :. :* **:** :

14128 260 -------GILYATDGT-IQIRDMMRWFKGT---NLVGKPKIFIFQACQGHEYMDGVDATDAPPADKR----------------------- 315
Casp9 241 ASHLQFPGAVYGTDGCPVSVEKIVNIFNGTSCPSLGGKPKLFFIQACGGEQKDHGFEVASTSPEDESPGSNPEPDATPFQEGLRTFDQLD 330
                 * :*.***  :.:..::. *:**   .* ****:*::*** *.:  .*.:.:.:.* *:

14128 316 --VQIPVEADFLYAYSTVPGYYSWRNSVNGSWFIQSIAEVFDKYAKTTDLLTMMTRVNALVATYQSRTNDPYSDRKKQIPSIVSMMRKDF 403
Casp9 331 AISSLPTPSDIFVSYSTFPGFVSWRDPKSGSWYVETLDDIFEQWAHSEDLQSLLLRVANAVSVKG---------IYKQMPGCFNFLRKKL 411
            .:*. :*:: :***.**: ***:. .***:::: ::*:::*:: ** ::: **   *:.             **:*. ..::**.:

14128 404 YFYPE 408
Casp9 412 FFKTS 416
          :* ..
```

B GENE23203 (Transcript 106327_1), 425 amino acids **Fig. S1**

Versus Human Caspase 3: 36% Identity over 262 amino acids

```
23203 161 YPMNRQPHGLCLIVNNATFEAESGLANRKGSHVDARNLQSLFLYLKYKVTVVENQRARVLKDTIMRFAKQDHSSYDSVIVCMLXHGLEGR 250
Casp3  37 YKMDYPEMGLCIIINNKNFHKSTGMTSRSGTDVDAANLRETFRNLKYEVRNKNDLTREEIVELMRDVSKEDHSKRSSFVCVLLSHGEEGI 126
          * *:    ***:*:** .*. .:*::.*.*:.*** **:. *  ***:*   ::   . : : :  .:*:***. .*.:  :* ** **

23203 251 IYGVDGGLVSINDLVSMFNGYNAKTLIGKPKLFFIQACRGGDFDRGITFERIDGLESSPLEEKPVEEVLEELYPSEEADSGFXAPQSLPS 340
Casp3 127 IFGTNG-PVDLKKITNFFRGDRCRSLTGKPKLFIIQACRGTELDCGIETD--SGVDDD------------------------MACHKIPV 189
          *:*.:*  *.::.:..:*.* ..::* ******:****** ::* **  : .*::..                        * :.:*

23203 342 EADILIAYXTVPGFVSWRHSDKGSWFVQALVXVFRQHSVNEDVASMLIKINRKVALEFESS------DKKKQMPAPXIMLTRKVFFFP 422
Casp3 190 EADFLYAYSTAPGYYSWRNSKDGSWFIQSLCAMLKQYADKLEFMHILTRVNRKVATEFESFSFDATFHAKKQIPCIVSMLTKELYFYH 277
          ***:* ** *.**: ***:*..****:*:*  :::*:: : :.  :* ::***** ****        . ***:*.   ***::::*:
```

Versus Human Caspase 7: 36% Identity over 262 amino acids

```
23203 161 YPMNRQPHGLCLIVNNATFEAESGLANRKGSHVDARNLQSLFLYLKYKVTVVENQRARVLKDTIMRFAKQDHSSYDSVIVCMLXHGLEGR 250
Casp7  60 YNMNFEKLGKCIIINNKNFDKVTGMGVRNGTDKDAEALFKCFRSLGFDVIVYNDCSCAKMQDLLKKASEEDHTNAACFACILLSHGEENV 149
          * ** :  * *:*:** .*:  :*:. *:*:. **. * . *  * :.* * ::  .  ::* : : :::**:.  ..   :* ** *.

23203 251 IYGVDGGLVSINDLVSMFNGYNAKTLIGKPKLFFIQACRGGDFDRGITFERIDGLESSPLEEKPVEEVLEELYPSEEADSGFXAPQSLPS 340
Casp7 150 IYGKDG-VTPIKDLTAHFRGDRCKTLLEKPKLFFIQACRGTELDDGIQAD------SGPIN-----------------DTDANPRYKIPV 215
          *** ** :..*:**.: *.* ..***: ************ ::* **  :       *.*::                  *:. .  .:*

23203 341 EADILIAYXTVPGFVSWRHSDKGSWFVQALVXVFRQHSVNEDVASMLIKINRKVALEFESSD------KKKQMPAPXIMLTRKVFFFP 422
Casp7 216 EADFLFAYSTVPGYYSWRSPGRGSWFVQALCSILEEHGKDLEIMQILTRVNDRVARHFESQSDDPHFHEKKQIPCVVSMLTKELYFSQ 303
          ***:*:** ****: *** ..:*********  ::.:*. : :: .:* ::* :** .***..      :***:*.   ***::::*
```

Versus Human Caspase 9: 37% Identity over 263 amino acids

```
23203 161 YPMNRQPHGLCLIVNNATFEAESGLANRKGSHVDARNLQSLFLYLKYKVTVVENQRARVLKDTIMRFAKQDHSSYDSVIVCMLXHG---- 246
Casp9 153 YILSMEPCGHCLIINNVNFCRESGLRTRTGSNIDCEKLRRRFSSPHFMVEVKGDLTAKKMVLALLELAQQDHGALDCCVVVILSHGCQAS 242
          * :. :* * ***:**..*   **** .*.**::*..:*:   *    :: * *  :   *: :  :::.:*:***.: *. :* :* **

23203 247 ---LEGRIYGVDGGLVSINDLVSMFNGYNAKTLIGKPKLFFIQACRGGDFDRGITFERIDGLESSPLEEKPVEEVLEELYPSEEADSGFX 333
Casp9 241 HLQFPGAVYGTDGCPVSVEKIVNIFNGTSCPSLGGKPKLFFIQACGGEQKDHGFEVASTSPEDESPGSNP--EPDATPFQEGLRTFDQLD 328
             : * :**.**  **::.:*.:*** .. :* *********** * : *:*: .    .  :.** .:    *      :  . .: . :

23203 334 APQSLPSEADILIAYXTVPGFVSWRHSDKGSWFVQALVXVFRQHSVNEDVASMLIKINRKVALEFESSDKKKQMPAPXIMLTRKVFFFPK 423
Casp9 329 AISSLPTPSDIFVSYSTFPGFVSWRDPKSGSWYVETLDDIFEQWAHSEDLQSLLLRVANAVSVKG----IYKQMPGCFNFLRKKLFFKTS 416
          * .***: :**:::* *.*******....***:*::*  :*.* : .**: *:*::: . *:::        ****.   :* :*:** ..
```

C GENE25497 (Transcript 54104_1), ≈ 436 amino acids

Versus Human Caspase 3: 32% Identity over 247 amino acids

```
25497 235 YPSNKRPRGICLIFNNKTFNQDRSLHLPRRDGTEKDVENLKLLWEKLHFXVIVKPDLTAHEMYDTARDYSRLDHSNYDCFVCCILSHGVQ 324
Casp3  37 YKMDYPEMGLCIIINNKNFHKSTGMTS--RSGTDVDAANLRETFRNLKYEVRNKNDLTREEIVELMRDVSKEDHSKRSSFVCVLLSHGEE 124
          *  :    *:*:*:***.*::. .:    *.**: *. **:  :.:*:: *  * *** .*: :  ** *: ***: ..*** :**** :

25497 325 GGIYGSDGEIIELGQITSQFKGTACXSLANKPKLFFVQACRGTDFDPGVQADAVXNSDEEAMRHSAEPNEGHFLLGYATPPGYVSWRSTQ 414
Casp3 125 GIIFGTNG-PVDLKKITNFFRGDRCRSLTGKPKLFIIQACRGTELDCGIETDSG---VDDDMACHKIPVEADFLYAYSTAPGYYSWRNSK 210
          * *:*::*  ::* :**. *:*  * **:.*****::******::* *:::*:      :: *      * *..** .*:*.*** ***.::

25497 415 HGSWYISHLCDVFHTYCERFDIMSMMVMVNGKVSEAYTKKGYKQCPAPVSPVNCLDSQEEETKSLYK 481
Casp3 211 DGSWFIQSLCAMLKQYADKLEFMHILTRVNRKVATEFESFSFDATFHAKKQIPCIVSMLTKELYFYH 277
          .***:*. ** ::: *.:::::* ::. ** **:  : . .:.     . . : *: *   :   :*:
```

Versus Human Caspase 7: 33% Identity over 249 amino acids

```
25497 235 YPSNKRPRGICLIFNNKTFNQDRSLHLPRRDGTEKDVENLKLLWEKLHFXVIVKPDLTAHEMYDTARDYSRLDHSNYDCFVCCILSHGVQ 324
Casp7  60 YNMNFEKLGKCIIINNKNFDKVTGMGV--RNGTDKDAEALFKCFRSLGFDVIVYNDCSCAKMQDLLKKASEEDHTNAACFACILLSHGEE 147
          *  * .  * *:*:***.*::  .: :   :**:**.* *   :..* * ***  * :. :* *  :. *. **:*   **.* :**** :

25497 325 GGIYGSDGEIIELGQITSQFKGTACXSLANKPKLFFVQACRGTDFDPGVQADAVXNSDEEAMRHSAEPNEGHFLLGYATPPGYVSWRSTQ 414
Casp7 148 NVIYGKDG-VTPIKDLTAHFRGDRCKTLLEKPKLFFIQACRGTELDDGIQADSGPINDTDANPRYKIPVEADFLFAYSTVPGYYSWRSPG 236
          . ***.** :  : ::*::*:*  * :* :******:******::* *:***:   .* :*  :    * *..**:.*:* *** ****.

25497 415 HGSWYISHLCDVFHTYCERFDIMSMMVMVNGKVSEAYTKKGYKQCPAPVSPVNCLDSQEEETKSLYKDP 483
Casp7 237 RGSWFVQALCSILEEHGKDLEIMQILTRVNDRVARHFESQSDDPHFHEKKQIPCVVS--MLTKELYFSQ 303
          :***::. **.::. : : ::**.::. **.:*:. : .:. .      . : *: *    **.** .
```

Versus Human Caspase 9: 37% Identity over 249 amino acids

```
25497 235 YPSNKRPRGICLIFNNKTFNQDRSLHLPRRDGTEKDVENLKLLWEKLHFXVIVKPDLTAHEMYDTARDYSRLDHSNYDCFVCCILSHGVQ 324
Casp9 153 YILSMEPCGHCLIINNVNFCRESGLRT--RTGSNIDCEKLRRRFSSPHFMVEVKGDLTAKKMVLALLELAQQDHGALDCCVVVILSHGCQ 240
          *  . .* * ***:** .* :: .*:   * *:: * *:*:  :.. ** * ** ****::*  :  : :: **.   ** *  ***** *

25497 325 G-------GIYGSDGEIIELGQITSQFKGTACXSLANKPKLFFVQACRGTDFDPGVQADAVXNSDEEAMRHSAEPNEGHFLLGYAT---- 403
Casp9 241 ASHLQFPGAVYGTDGCPVSVEKIVNIFNGTSCPSLGGKPKLFFIQACGGEQKDHGFEVAST-SPEDESPGSNPEPDATPFQEGLRTFDQL 329
          .       .:**:**  :.: :*.. *:**:* **..******:*** * : * *.:. :. ..::*:   ..**:   *  *  *

25497 404 --------P----------PGYVSWRSTQHGSWYISHLCDVFHTYCERFDIMSMMVMVNGKVSEAYTKKGYKQCPAPVSPVNCLDSQEEE 475
Casp9 330 DAISSLPTPSDIFVSYSTFPGFVSWRDPKSGSWYVETLDDIFEQWAHSEDLQSLLLRVANAVS---VKGIYKQMPG------CFNFLR-- 408
                  *          **:****..: ****:. * *:*. :..  *: *::: * . **   .*  *** *.      *::  .

25497 476 TKSLYKDP 483
Casp9 409 KKLFFKTS 416
          .* ::* .
```
