# Supplementary figures and images for: Relative Contributions of Various Cellular Mechanisms to Loss of Algae during Cnidarian Bleaching
Source: PLoS One. 2016 Apr 27;11(4):e0152693. doi: 10.1371/journal.pone.0152693 (PMC4847765; doi:10.1371/journal.pone.0152693)

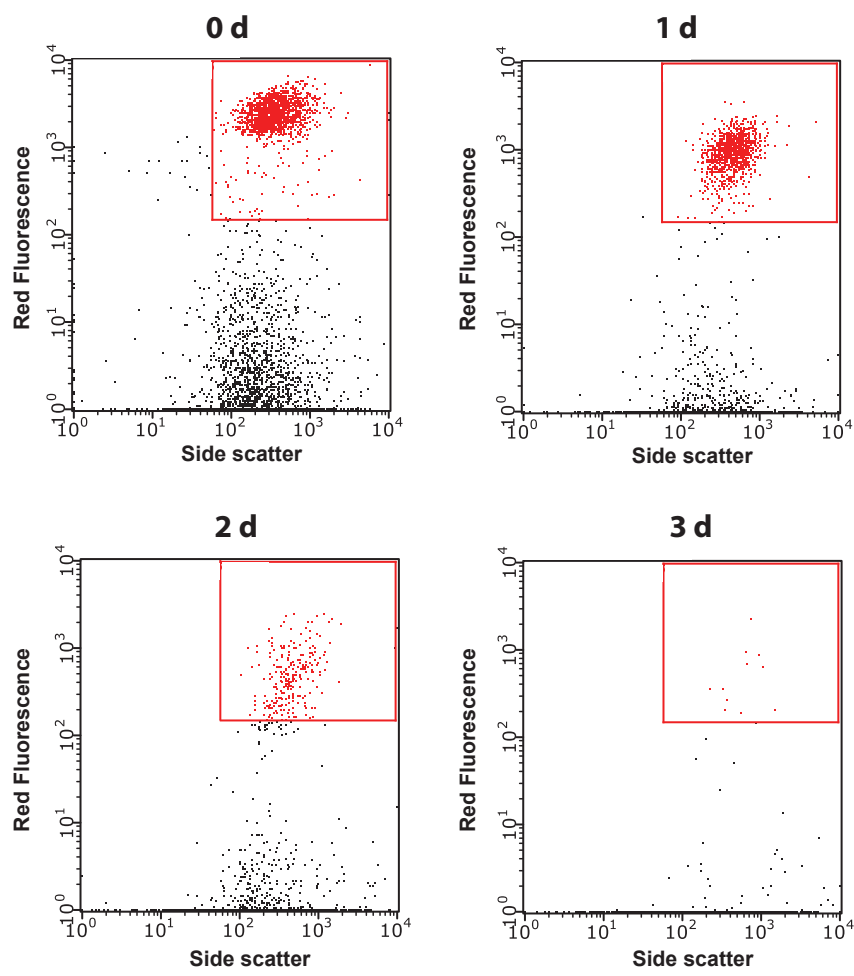

Supplement: S2 Fig — Red chlorophyll fluorescence is plotted against side scatter (a measure of particle size and refractive index) for particles passing through the laser beam in the flow cytometer. The red square denotes the window in which Symbiodinium cells normally fall. Additional details are provided elsewhere [51]. (PDF) [file pone.0152693.s002.pdf]

27°C, 25  $\mu\text{mol photons m}^{-2} \text{s}^{-1}$

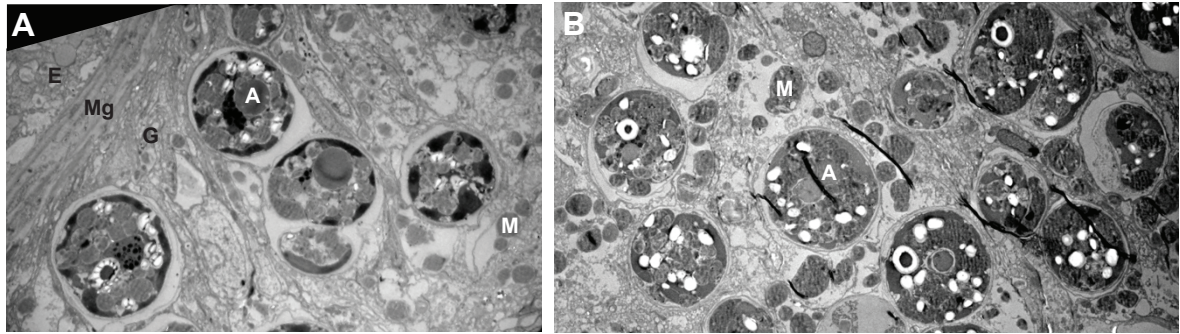

34°C, 150  $\mu\text{mol photons m}^{-2} \text{s}^{-1}$

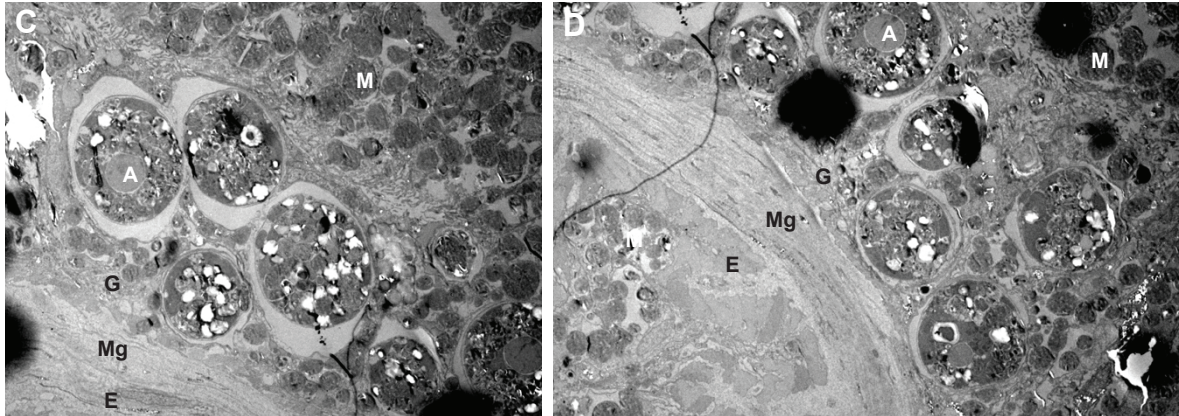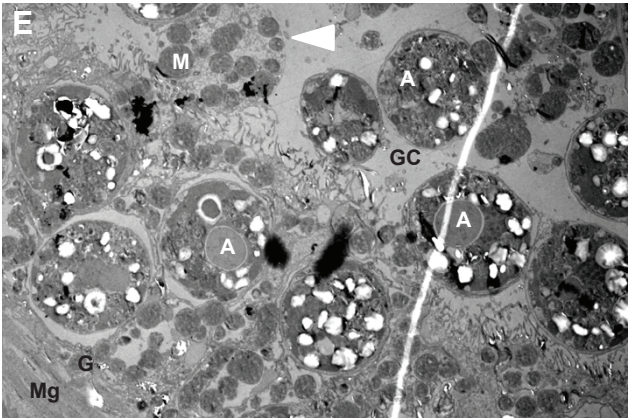

10  $\mu\text{m}$

Supplement: S3 Fig — In an experiment like that of Fig 2C and 2D, symbiotic anemones were fixed after collection from populations under standard culture conditions (A,B) or after 6 d of heat-and-light stress (C-E). Sections were then examined by electron microscopy as described in Materials and Methods. A and B are sections of different anemones; C-E are different fields of view in the same anemone section. A, alga; E, epidermis; G, gastrodermis; GC, gastric cavity; M, mitochondria; Mg, mesoglea. Scale bar (all panels), 10 μm. In E, conspicuous white line is a sectioning artifact, and the arrowhead indicates what may be either a detached host cell in the gastric cavity or an attached cell seen in this section because of distortion during embedding and sectioning. Note also seemingly free algal cells in the gastric cavity. (PDF) [file pone.0152693.s003.pdf]

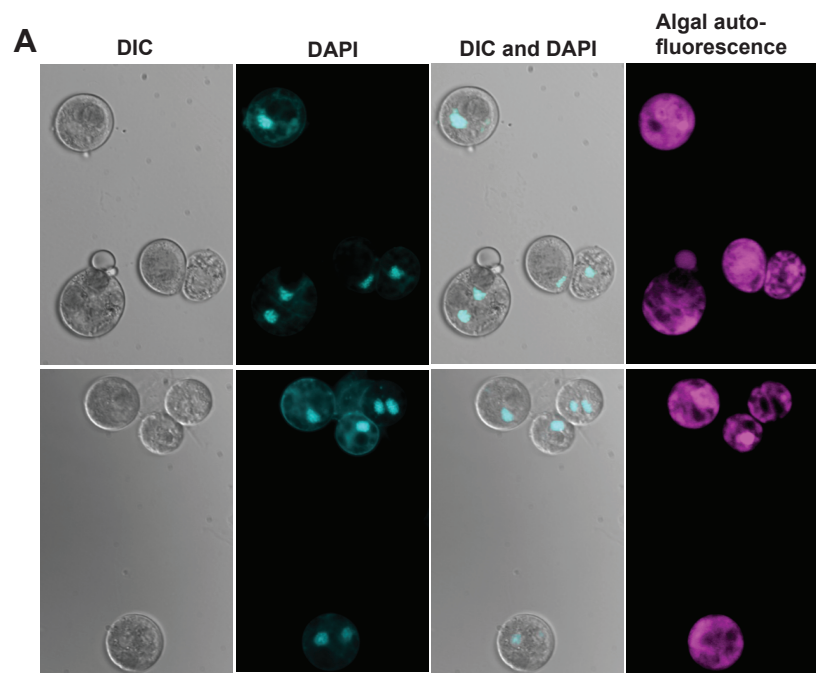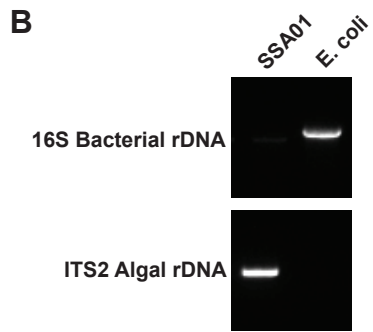

Supplement: S4 Fig — (A) SSA01 cells (see Materials and Methods) were grown in liquid IMK medium containing both casein hydrolysate and glucose (so that heterotrophic contaminants could grow), stained with DAPI, and examined by DIC and fluorescence microscopy as described in Materials and Methods. Scale bar, 5 μm. (B) DNA from the cultured cells was used as template for attempted amplification of bacterial 16S rDNA by PCR (see Materials and Methods). Amplification of the 16S fragment from E. coli (using the same primers and conditions) and of the Symbiodinium rDNA ITS2 region served as controls. (PDF) [file pone.0152693.s004.pdf]
